# Giant magnetochiral anisotropy from quantum-confined surface states of topological insulator nanowires

**DOI:** 10.1038/s41565-022-01124-1

**Published:** 2022-05-12

**Authors:** Henry F. Legg, Matthias Rößler, Felix Münning, Dingxun Fan, Oliver Breunig, Andrea Bliesener, Gertjan Lippertz, Anjana Uday, A. A. Taskin, Daniel Loss, Jelena Klinovaja, Yoichi Ando

**Affiliations:** 1 Department of Physics, University of Basel, Basel, Switzerland; 2 Physics Institute II, University of Cologne, Cologne, Germany; 3 Quantum Solid State Physics, KU Leuven, Leuven, Belgium

**Keywords:** Electronic devices, Topological insulators, Electronic properties and materials, Electronic and spintronic devices

## Abstract

Wireless technology relies on the conversion of alternating electromagnetic fields into direct currents, a process known as rectification. Although rectifiers are normally based on semiconductor diodes, quantum mechanical non-reciprocal transport effects that enable a highly controllable rectification were recently discovered^1–9^. One such effect is magnetochiral anisotropy (MCA)^6–9^, in which the resistance of a material or a device depends on both the direction of the current flow and an applied magnetic field. However, the size of rectification possible due to MCA is usually extremely small because MCA relies on inversion symmetry breaking that leads to the manifestation of spin–orbit coupling, which is a relativistic effect^6–8^. In typical materials, the rectification coefficient *γ* due to MCA is usually ∣*γ*∣ ≲ 1 A^−1^ T^−1^ (refs. ^8–12^) and the maximum values reported so far are ∣*γ*∣ ≈ 100 A^−1^ T^−1^ in carbon nanotubes^13^ and ZrTe_5_ (ref. ^14^). Here, to overcome this limitation, we artificially break the inversion symmetry via an applied gate voltage in thin topological insulator (TI) nanowire heterostructures and theoretically predict that such a symmetry breaking can lead to a giant MCA effect. Our prediction is confirmed via experiments on thin bulk-insulating (Bi_1−*x*_Sb_*x*_)_2_Te_3_ (BST) TI nanowires, in which we observe an MCA consistent with theory and ∣*γ*∣ ≈ 100,000 A^−1^ T^−1^, a very large MCA rectification coefficient in a normal conductor.

## Abstract (web summary)

Non-reciprocal transport in a homogeneous material enables controllable current rectification, but is usually very small. Yet, artificially breaking inversion symmetry in topological insulator nanowires yields a giant magnetochiral anisotropy rectification.

## Main

In most materials transport is well described by Ohm’s law, *V* = *IR*_0_, dictating that for small currents *I* the voltage drop across a material is proportional to a constant resistance *R*_0_. Junctions that explicitly break inversion symmetry, for instance semiconductor *pn* junctions, can produce a difference in resistance *R* as a current flows in one or the opposite direction through the junction, *R*( + *I*) ≠ *R*( − *I*); this difference in resistance is the key ingredient required to build a rectifier. A much greater degree of control over the rectification effect can be achieved when a similar non-reciprocity of resistance exists as a property of a material rather than a junction. However, to achieve such a non-reciprocity necessitates that the inversion symmetry of the material is itself broken. Previously, large non-reciprocal effects were observed in materials where inversion symmetry breaking resulted in strong spin–orbit coupling (SOC)^6–12,14^. However, as SOC is always a very small energy scale, this limits the possible size of any rectification effect.

The non-reciprocal transport effect considered here is magnetochiral anisotropy (MCA), which occurs when both inversion and time-reversal symmetry are broken^6–12,14^. When allowed, the leading order correction of Ohm’s law due to MCA is a term that is second order in current and manifests itself as a resistance of the form *R* = *R*_0_(1 + *γBI*), with *B* the magnitude of an external magnetic field and the rectification coefficient *γ* determines the size of the possible rectification effect. MCA may also be called bilinear magnetoelectric resistance^9,15^. We note that non-reciprocal transport in ferromagnets^3,4^ does not allow the coefficient *γ* to be calculated and rectification of light into d.c. current due to bulk photovoltaic effects^16–18^ concerns much higher energy scales than those of MCA.

**Fig. 1:**
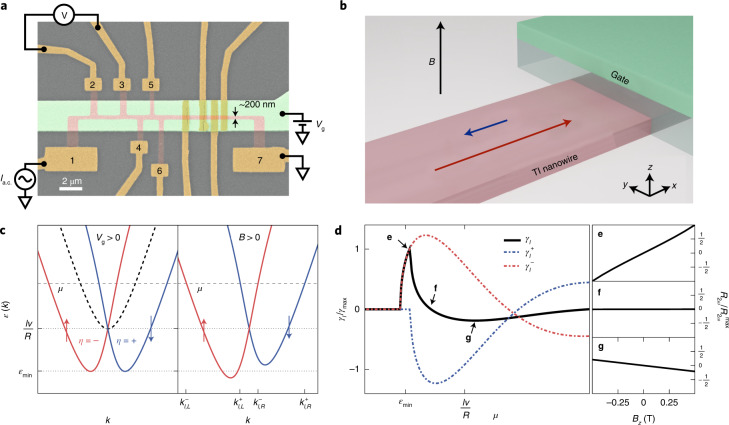
Gate-tunable TI nanowire device and the theory of MCA. **a**, False-colour scanning electron microscope image of device 1 with schematics of the electrical wiring; the Pt/Au leads are in dark yellow, the TI nanowire etched from an MBE-grown BST thin film are in red and the top-gate electrode are in green. The resistance of the nanowire was measured on different sections: sections 1, 2, 3, 4 and 5 correspond to the voltage contact pairs 2–3, 3–4, 4–5, 5–6 and 2–6 (the numbers shown), respectively. **b**, Schematic of MCA in TI nanowires. A gate, applied here to the top of the nanowire, breaks the inversion symmetry along the wire. Applying a magnetic field along the gate normal (*z* direction) results in a giant MCA rectification such that current flows more easily in one direction along the wire than in the opposite one (indicated by red and blue arrows, respectively). **c**, Energy dispersion *ε*(*k*) of the TI nanowire surface states, which form degenerate subbands (dashed line). When a finite *V*_g_ is applied, the inversion symmetry is broken and the subbands split (solid lines). A new minimum subband energy occurs at *ε*_min_ and the states possess a finite spin polarization in the *yz* plane (red and blue lines indicate subbands with opposite spin polarization). A magnetic field *B* shifts the subband pair relative to each other in terms of energy due to the Zeeman effect, which is maximal for *B* along the *z* axis and leads to an MCA (the size of the shift shown here is used for clarity and is not to scale). **d**, Size of the MCA rectification *γ*_*l*_ (equation (1)) as a function of chemical potential *μ* within a given subband pair. Owing to the peculiar dispersion of a TI nanowire, the curvature, ${\hslash }^{2}{{{V}}}_l^{\eta }(k)\equiv {\partial }_{k}^{2}{\varepsilon }_l^{\eta }(k)$, is large and highly anisotropic at opposite Fermi momenta, which results in a giant MCA. As the chemical potential *μ* is tuned from the bottom of the subband, *γ*_*l*_ changes sign. Here, for clarity, we used *B* = 1 T (see Supplementary Note 5 for further parameters). **e**–**g**, The theoretically expected magnetic-field dependence of *R*_2*ω*_ at the chemical potentials indicated in **d**.

In heterostructures of topological materials it is possible to artificially break the inversion symmetry of a material^19^; such an approach provides an unexplored playground to substantially enhance the size of non-reciprocal transport effects. In this context, quasi-one-dimensional (1D) bulk-insulating 3D TI nanowires^19–23^ are the perfect platform to investigate large possible MCAs due to artificial inversion symmetry breaking. In the absence of symmetry breaking, for an idealized cylindrical topological insulator (TI) nanowire—although generalizable to an arbitrary cross-section^19,22^—the surface states form energy subbands of momentum *k* along the nanowire and half-integer angular momentum $l =\pm \frac{1}{2},\frac{3}{2},\ldots \,$ around the nanowire, where the half-integer values are due to spin-momentum locking. The presence of inversion symmetry along a TI nanowire requires the subbands with angular momenta ±*l* to be degenerate. It is possible to artificially break the inversion symmetry along the wire, for instance, by the application of a gate voltage from the top of the TI nanowire^19,21,23^. Such a gate voltage induces a non-uniformity of charge density across the nanowire cross-section, which breaks the subband degeneracy and results in a splitting of the subband at finite momenta^19^ (Fig. 1c). An additional consequence is that the subband states develop a finite spin polarization in the plane perpendicular to the nanowire axis (that is, the *yz* plane) with the states with opposite momenta being polarized in the opposite directions, such that the time-reversal symmetry is respected. When a magnetic field is applied, the subbands can be shifted in energy via the Zeeman effect, which suggests that an MCA can be present in this set-up. Indeed, using the Boltzmann equation^10,11,14^ (Supplementary Note 4), we found an MCA of the vector-product type $\gamma \propto {{{{{\mathbf{P}}}}}}\cdot (\hat{{{{{{\mathbf{B}}}}}}}\times \hat{{{{{{\mathbf{I}}}}}}})$ with the characteristic vector **P** in the *yz* plane. For the rectification effect *γ*_*l*_(*μ*) of a given subband pair *η* = ± labelled by *l* > 0, we found:

1 $${\gamma }_l={\gamma }_l^{+}+{\gamma }_l^{-}\approx \frac{{e}^{3}}{{({\sigma }^{(1)})}^{2}hB}\mathop{\sum}\limits_{\eta =\pm }{\tau }^{2}\left[{{{V}}}_l^{\eta }({k}_{l,{\rm{R}}}^{\eta })-{{{V}}}_l^{\eta }({k}_{l,{\rm{L}}}^{\eta })\right],$$

where *e* is the elementary charge, *h* is the Planck constant, *σ*^(1)^ is the conductivity in linear response, *τ* is the scattering time, ${{{V}}}_l^{\eta }(k)=\frac{1}{{\hslash }^{2}}{\partial }_{k}^{2}{\varepsilon }_l^{\eta }(k)$ in which ${\varepsilon }_l^{\eta }(k)$ describes the energy spectrum as a function of momentum *k* in the presence of symmetry breaking terms and the finite magnetic field *B* (Fig. 1c and Supplementary Note 4) and ${k}_{l,{\rm{R}}({\rm{L}})}^{\eta }$ is the right (left) Fermi momentum of a given subband (Fig. 1c). Owing to the non-parabolic spectrum of the subbands, differences in ${{{V}}}_{l }^{\eta }(k)$ are large for a TI nanowire, which results in the giant MCA. The quantities ${\gamma }_{l }^{+}$ and ${\gamma }_{l }^{-}$ are the contributions of the individual subbands. The behaviour of *γ*_*l*_ as a function of the chemical potential *μ* is shown in Fig. 1d. We found that, as the chemical potential is tuned through the subband pair, *γ*_*l*_ changes sign depending on the chemical potential. This makes the rectification effect due to the MCA highly controllable by both the magnetic field direction and the chemical potential *μ* within a given subband pair, which can be experimentally adjusted by a small change in gate voltage *V*_g_. For reasonable experimental parameters, we predict that the theoretical size of the rectification can easily reach giant values of *γ* ≈ 5 × 10^5^ T^−1^ A^−1^ (Supplementary Note 5).

**Fig. 2:**
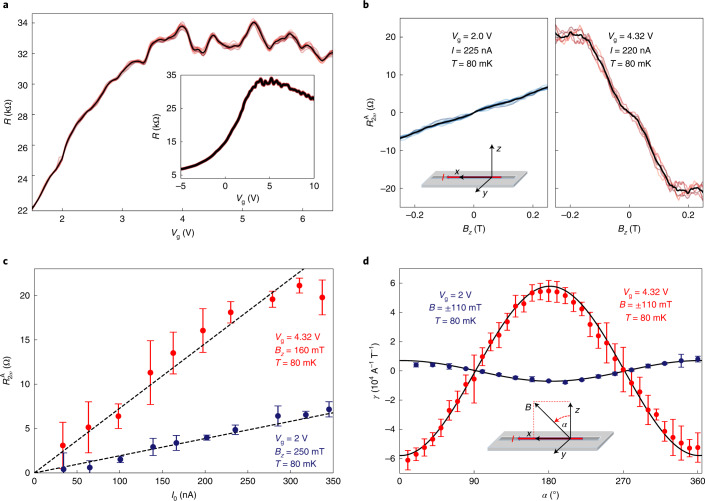
Non-reciprocal transport in BST TI nanowire. **a**, Four-terminal resistance *R* measured on device 1, section 1, at 30 mK in 0 T as a function of *V*_g_ showing reproducible peaks and dips around the resistance maximum, which are consistent with the response expected from quantum-confined surface states^23^. As the *R* value is very sensitive to the details of the charge distributions in or near the nanowires, the *R*(*V*_g_) behaviour is slightly different for different sweeps; thin red lines show the results of 15 unidirectional *V*_g_ sweeps and the thick black line shows their mean average. Inset: data for a wider range of *V*_g_, which demonstrates the typical behaviour of a bulk-insulating TI. **b**, Antisymmetric component of the second-harmonic resistance, ${R}_{2\omega }^{{\rm{A}}}$, for *V*_g_ = 2 and 4.32 V plotted versus a magnetic field *B* applied along the *z* direction (the coordinate system is depicted in the inset); coloured thin lines show ten (six) individual *B*-field sweeps for 2 V (4.32 V), and the thick black line shows their mean. **c**, ${R}_{2\omega }^{{\rm{A}}}$ measured for *V*_g_ = 2 and 4.32 V in the *B* field (applied in the *z* direction) of 0.25 and 0.16 T, respectively, as a function of the a.c. excitation current *I*_0_. The dashed lines are a guide to the eye to show the linear behaviour. Error bars are defined using the standard deviation of ten (six) individual *B*-field sweeps for 2 V (4.32 V). **d**, Magnetic-field-orientation dependencies of *γ* at *V*_g_ = 2 and 4.32 V when the *B* field is rotated in the *zx* plane. Error bars are defined using the minimum–maximum method with six (eight) individual *B*-field sweeps for 2 V (4.32 V). Solid black lines are the fits to *γ* ≈ *γ*_0_cos*α* expected for MCA. Inset: the definition of *α* and the coordinate system.

To experimentally investigate the predicted non-reciprocal transport behaviour, we fabricated nanowire devices^24^ of the bulk-insulating TI material BST, as shown in Fig. 1a by etching high-quality thin films grown by molecular beam epitaxy (MBE). The nanowires have a rectangular cross-section of thickness *d* ≈ 16 nm and width *w* ≈ 200 nm, with channel lengths up to several micrometres. The long channel lengths suppress coherent transport effects, such as universal conductance fluctuations, and the cross-sectional perimeter allows for the formation of well-defined subbands (Supplementary Note 8). An electrostatic gate electrode is placed on top of the transport channel for the dual purpose of breaking inversion symmetry and tuning the chemical potential. The resistance *R* of the nanowire shows a broad maximum as a function of *V*_g_ (Fig. 2a inset), which indicates that the chemical potential can be tuned across the charge neutrality point (of the surface-state Dirac cone; the dominant surface transport in these nanowires is further documented in Supplementary Note 7). Near the broad maximum (that is, around the charge neutrality point), the *V*_g_ dependence of *R* shows reproducible peaks and dips (Fig. 2a), which is a manifestation of the quantum-confined quasi-1D subbands realized in TI nanowires^23^—each peak corresponds to the crossing of a subband minima, although the feature can be smeared by disorder^23^. To measure the non-reciprocal transport, we used a low-frequency a.c. excitation current *I* = *I*_0_sin*ωt* and probed the second-harmonic resistance *R*_2*ω*_; here, *I*_0_ is the amplitude of the excitation current, *ω* is the angular frequency, and *t* is time. The MCA causes a second-harmonic signal that is antisymmetric with the magnetic field *B* and therefore we calculated the antisymmetric component ${R}_{2\omega }^{{\rm{A}}}\equiv \frac{{R}_{2\omega }({{{{{B}}}}})-{R}_{2\omega }(-{{{{{B}}}}})}{2}$, which is proportional to *γ* via ${R}_{2\omega }^{{\rm{A}}}=\frac{1}{2}\gamma {R}_{0}B{I}_{0}\approx \frac{1}{2}\gamma RB{I}_{0}$, where *R*_0_ is the reciprocal resistance (see Methods for details).

In our experiment, we observed a large ${R}_{2\omega }^{{\rm{A}}}$ for *V*_g_ ≳ 2 V with a magnetic field along the *z* axis. The ${R}_{2\omega }^{{\rm{A}}}({B}_{z})$ behaviour was linear for small *B*_*z*_ values (Fig. 2b) and ${R}_{2\omega }^{A}$ increased linearly with *I*_0_ up to ~250 nA (Fig. 2c), both of which are the defining characteristics of the MCA. The deviation from the linear behaviour at higher *B* fields is probably due to orbital effects (Supplementary Note 3). The magnetic-field-orientation dependence of *γ*, shown in Fig. 2d for the rotation in the *zx* plane, agrees well with *γ* ≈ *γ*_0_cos*α*, with *α* the angle from the *z* direction and *γ*_0_ the value at *α* = 0; the rotation in the *yz* plane gave similar results, whereas MCA remained essentially zero for the rotation in the *xy* plane (Supplementary Note 10). This points to the vector-product type MCA, ${R}_{2\omega }^{{\rm{A}}}\propto {{{{{\mathbf{P}}}}}}\cdot ({{{{{\mathbf{B}}}}}}\times {{{{{\mathbf{I}}}}}})$, with the characteristic vector **P** essentially parallel to *y*, which is probably dictated by the large *g*-factor anisotropy^25^ (Supplementary Note 2). The maximum size of the ∣*γ*∣ in Fig. 2d reaches a giant value of ∣*γ*∣ ≈ 6 × 10^4^ A^−1^ T^−1^. In addition, one may notice in Fig. 2b,d that the relative sign of *γ* changes for different *V*_g_ values, which is very unusual. We observed a giant MCA with a similarly large rectification *γ* in all the measured devices, some of which reached ~1 × 10^5^ A^−1^ T^−1^ (Supplementary Note 13). Note that in the MCA literature, *γ* is often multiplied by the cross-sectional area *A* of the sample to give *γ*′ (= *γA*), which is useful to compare the MCA in different materials as a bulk property. However, in nanodevices, such as our TI nanowires, the large MCA owes partly to mesoscopic effects and *γ*′ is not very meaningful. In fact, the large MCA rectification of ∣*γ*∣ ≈ 100 A^−1^ T^−1^ observed in chiral carbon nanotubes^13^ was largely due to the fact that a nanotube can be considered a quasi-1D system. In Supplementary Note 13, we present extensive comparisons of the non-reciprocal transport reported for various systems.

**Fig. 3:**
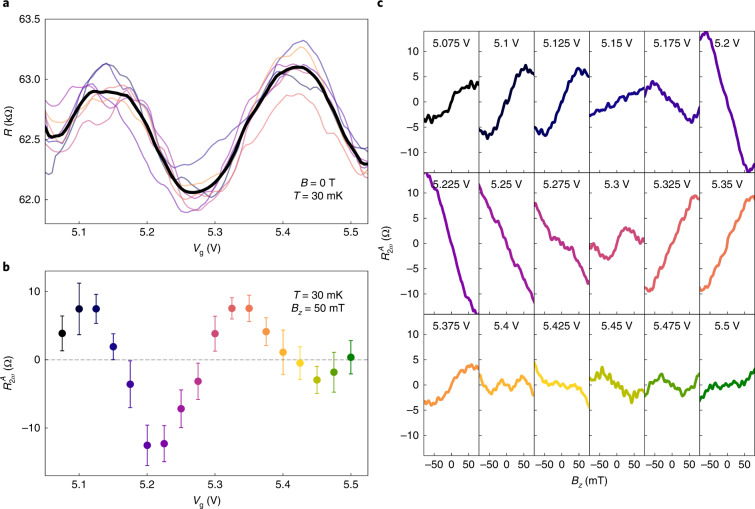
Evolution of the non-reciprocal response with changing chemical potential. **a**, *R* versus *V*_g_ data of device 3, section 5, in a narrow range of *V*_g_, in which the chemical potential was changed near the charge neutrality point (for a wider range of *V*_g_, see Supplementary Fig. 11a). The peaks in *R* occur when the bottom of one of the quantum-confined subbands is crossed by the chemical potential; the coloured thin lines show seven individual *V*_g_ sweeps and the thick black line shows their mean. **b**, Mean ${R}_{2\omega }^{{\rm{A}}}$ values at *B*_*z*_ = 50 mT for various gate voltages in the range corresponding to that in **a**. The zero crossings of ${R}_{2\omega }^{{\rm{A}}}$ roughly correspond to the peaks and dips in *R*(*V*_g_), and thereby are linked to the quantum-confined subbands. Error bars are defined using the standard deviation of ten individual *B*-field sweeps. **c**, Averaged ${R}_{2\omega }^{{\rm{A}}}({B}_{z})$ curves at various *V*_g_ settings, from which the data points in **b** were calculated (data points and curves are coloured correspondingly). The systematic change in the ${R}_{2\omega }^{{\rm{A}}}({B}_{z})$ behaviour as a function of *V*_g_ is clearly visible.

A unique feature of the predicted MCA is the controllability of its sign with a small change of *V*_g_. To confirm this prediction, we measured detailed *V*_g_ dependences of ${R}_{2\omega }^{{\rm{A}}}$ in the *V*_g_ range of 5.1–5.5 V, in which the chemical potential appears to pass through two subband minima, because *R*(*V*_g_) presents two peaks (Fig. 3a). We, indeed, observed the slope of ${R}_{2\omega }^{{\rm{A}}}({B}_{z})$ to change sign with *V*_g_ (Fig. 3b), and its zero-crossing roughly coincides with the peak or dip in the *R*(*V*_g_) curve (compare Fig. 3a,b). A change in sign of the slope of ${R}_{2\omega }^{A}({B}_{z})$ on either side of the *R*(*V*_g_) peaks was also observed in other devices (Supplementary Note 11). To obtain confidence in this striking observation, the evolution of the ${R}_{2\omega }^{{\rm{A}}}({B}_{z})$ behaviour on changing *V*_g_ is shown in Fig. 3c for many *V*_g_ values. This sign change on a small change of *V*_g_ also endows the giant MCA in TI nanowires with an unprecedented level of control. In addition, this *V*_g_-dependent sign change of MCA gives a unique proof that the origin of the peak-and-dip feature in *R*(*V*_g_) is, indeed, subband crossings.

The giant MCA observed here due to an artificial breaking of inversion symmetry in the TI nanowires not only results in a maximum rectification coefficient *γ* that is extremely high, but it is also highly controllable by small changes of chemical potential. Although rather different to the MCA of a normal conductor discussed here, we note that large rectification effects of a similar magnitude were recently discovered in non-centrosymmetric superconductor devices^1,5^ and in quantum anomalous Hall edge states^4^, for which the controllability is comparatively limited. It is prudent to mention that the MCA reported here was measured below 0.1 K and it diminishes at around 10 K (Supplementary Note 12), which is consistent with the sub-bandgap of ~1 meV. As TI nanowire devices are still in their infancy^24^, the magnitude and temperature dependence of the MCA could be improved with future improvements in nanowire quality and geometry; for example, in a 20-nm-diameter nanowire, the sub-bandgap would be ~10 meV, which enables MCAs up to ~100 K. The presence of the giant MCA provides compelling evidence for a large spin splitting of the subbands in TI nanowires with a broken inversion symmetry, which can be used for spin filters^26,27^. Moreover, it has been suggested that the helical spin polarization and large energy scales possible in such TI nanowires with a broken inversion symmetry can be used as a platform for robust Majorana bound states^19^, which are an integral building block for future topological quantum computers.

## Methods

### Theory

Transport coefficients were calculated using the Boltzmann equation^11,14^ to attain the current density due to an electric field *E* up to the second order such that *j* = *j*^(1)^ + *j*^(2)^ = *σ*^(1)^*E* + *σ*^(2)^*E*^2^. As discussed in ref. ^11^, experimentally the voltage drop *V* = *EL* as a function of current *I* is measured in the form *V* = *R*_0_*I*(1 + *γBI*). Using *R*_0_ = *L*/*σ*^(1)^ for a nanowire of length *L*, a comparison with the experimental behaviour can then be achieved via the relation ${\gamma }_{0}=-\frac{{\sigma }^{(2)}}{B{({\sigma }^{(1)})}^{2}}$. Although the linear response conductivity *σ*^(1)^ contains small peaks and dips due to an increased scattering rate close to the bottom of a subband, such fluctuations occur on top of a large constant conductivity and we therefore approximate ${\gamma }_{0}\approx \frac{A}{B}{\sigma }^{(2)}$, with $A=-1/{({\sigma }^{(1)})}^{2}$ approximately constant.

### Material growth and device fabrication

A 2 × 2 cm^2^ thin film of BST was grown on a sapphire (0001) substrate by co-evaporation of high-purity Bi, Sb and Te in an ultrahigh vacuum MBE chamber. The flux of Bi and Sb was optimized to obtain the most bulk-insulating film, which was achieved with a ratio of 1:6.8. The thickness varied in the range 14–19 nm in the whole film. Immediately after taking the film out of the MBE chamber, it was capped with a 3-nm-thick Al_2_O_3_ capping layer grown by atomic-layer deposition at 80 °C using an Ultratec Savannah S200. The carrier density and the mobility of the film were extracted from Hall measurements performed at 2 K using a Quantum Design PPMS. Gate-tunable multiterminal nanowire devices were fabricated using the following top-down approach: after defining the nanowire pattern with electron-beam lithography, the film was first dry etched using a low-power Ar plasma and then wet etched with a H_2_SO_4_/H_2_O_2_/H_2_O aqueous solution. To prepare the contact leads, the Al_2_O_3_ capping layer was removed in a heated aluminium etchant (Type-D, Transene) and 5/45 nm Pt/Au contacts were deposited by ultrahigh vacuum sputtering. Then, the whole device was capped with a 40-nm-thick Al_2_O_3_ dielectric grown by atomic-layer deposition at 80 °C, after which the 5/40 nm Pt/Au top gate was sputter deposited. Scanning electron microscopy was used to determine the nanowire size. Devices 1–4 reported in this Letter were fabricated on the same film in one batch, whereas device 5 (Supplementary Notes 7 and 8) was fabricated on a similar film.

### Second-harmonic resistance measurement

Transport measurements were performed in a dry dilution refrigerator (Oxford Instruments TRITON 200, base temperature ~20 mK) equipped with a 6/1/1-T superconducting vector magnet. The first- and second-harmonic voltages were measured in a standard four-terminal configuration with a low-frequency lock-in technique at 13.37 Hz using NF Corporation LI5645 lock-ins. In the presence of the vector-product-type MCA with ${{{\bf{P}}}}\parallel \hat{{{{\boldsymbol{y}}}}}$, the voltage is given by *V* = *R*_0_*I*(1 + *γBI*) for ${{{\bf{I}}}}\parallel \hat{{{{\boldsymbol{x}}}}}$ and ${{{\bf{B}}}}\parallel \hat{{{{\boldsymbol{z}}}}}$, where a hat indicates a unit vector in the given direction. For an a.c. current *I* = *I*_0_sin*ωt* this becomes $V={R}_{0}{I}_{0}\sin \omega t+\frac{1}{2}\gamma {R}_{0}B{I}_{0}^{2}[1+\sin (2\omega t-\frac{\uppi }{2})]$, which allows us to identify ${R}_{2\omega }=\frac{1}{2}\gamma {R}_{0}B{I}_{0}$ by measuring the out-of-phase component of the a.c. voltage at a frequency of 2*ω*. The d.c. gate voltage was applied using a Keithley 2450.

### Error bars

In the plots of ${R}_{2\omega }^{A}$ versus *I* shown in Fig. 2c (and in Supplementary Figs. 9b, 10b, 11b and 12b), the data points for each current value were calculated by obtaining slopes from linear fits to the ${R}_{2\omega }^{{\rm{A}}}(B)$ data at that current in the indicated *B* range (done individually for each measured *B* sweep); the standard deviation was calculated for the set of obtained slopes at each current and used as the error bar. In the plots of *γ* versus the angle shown in Fig. 2d (and in Supplementary Figs. 6 and 7) as well as the plot of *γ* versus *T* shown in Supplementary Fig. 13, the data points for each angle were calculated by obtaining slopes from linear fits to the ${R}_{2\omega }^{{\rm{A}}}(B)$ data at that angle in the indicated *B* range (done individually for each measured *B* sweep); from the set of obtained slopes at each angle, the error was calculated by using a minimum–maximum approach, in which we calculate the error to be half of the difference between the maximum and the minimum (calculating the standard deviation gives very similar results). In the plots of ${R}_{2\omega }^{{\rm{A}}}$ versus *V*_g_ shown Fig. 3b, the data points for each *V*_g_ value were calculated by obtaining slopes from the linear fits to the ${R}_{2\omega }^{{\rm{A}}}(B)$ data (shown in Fig. 3c) at that *V*_g_ in the indicated *B* range (done individually for each measured *B* sweep); from the set of obtained slopes per *V*_g_, the standard deviation was calculated and used as the error bar.

## Online content

Any methods, additional references, Nature Research reporting summaries, source data, extended data, supplementary information, acknowledgements, peer review information; details of author contributions and competing interests; and statements of data and code availability are available at 10.1038/s41565-022-01124-1.

## Supplementary information

Supplementary Information Supplementary Figs. 1–14, Notes 1–13 and Tables 1 and 2.

## Data Availability

The data that support the findings of this study are available at the online depository figshare with the identifier 10.6084/m9.figshare.19336571^28^ and Supplementary Information. Additional data are available from the corresponding authors upon reasonable request.
