# Supplementary material for: Giant magnetochiral anisotropy from quantum-confined surface states of topological insulator nanowires
Source: Nat Nanotechnol. 2022 May 12;17(7):696–700. doi: 10.1038/s41565-022-01124-1 (PMC9300460; doi:10.1038/s41565-022-01124-1)
Supplement: Supplementary file 1 — Supplementary Figs. 1–14, Notes 1–13 and Tables 1 and 2. [file 41565_2022_1124_MOESM1_ESM.pdf]

nature portfolio

https://doi.org/10.1038/s41565-022-01124-1

Supplementary information

Giant magnetochiral anisotropy from quantum-confined surface states of topological insulator nanowires

In the format provided by the authors and unedited

Supplementary Information for "Giant magnetochiral anisotropy from quantum confined surface states of topological insulator nanowires"

THEORETICAL DESCRIPTIONS

Supplementary Note 1. Model Hamiltonian and symmetries

Since the nanowires considered in our experiments are etched from thin films of $(Bi_{1-x}Sb_x)_2Te_3$, the exact crystallographic direction of the nanowire is unknown and therefore in general the only remaining symmetries [1] of $(Bi_{1-x}Sb_x)_2Te_3$ are inversion symmetry ($\mathcal{I}: x \to -x, y \to -y, z \to -z$) and time-reversal symmetry (represented as $\Theta = i\sigma_y K$, where K is complex conjugation and σ_i are Pauli-matrices operating in spin space). To linear order a general Hamiltonian satisfying these symmetries – for analytic simplicity we will use a cylindrical nanowire – is given by

$$H(x,\theta) = \frac{v_F}{2r} - iv_F\left[\sigma_x\partial_\theta/R - \{\cos\varphi(\sigma_y\cos\theta - \sigma_z\sin\theta) + \sin\varphi(\sigma_y\sin\theta + \sigma_z\cos\theta)\}\,\partial_x\right], \quad (1)$$

where $\partial_\theta/R = \cos(\theta)\partial_y - \sin(\theta)\partial_z$, $2\pi r$ is the nanowire perimeter, v_F is the Fermi-velocity, and θ is the angle measured from the top of the nanowire. The angle φ parameterises the direction of the spin-texture perpendicular to the wire axis. In the [111] surface states of a bulk TI, an out-of-plane component ($\varphi \neq 0$) can occur, for instance, due to hexagonal warping effect [2]; in a nanowire, however, the exact amount of rotation is theoretically undetermined due to the low symmetry involving the side surface, and it will be determined by the experiment.

It can be see the Hamiltonian Eq. (1) has inversion symmetry since $H(x,\theta) = H(-x,\theta+\pi)$ and also obeys time-reversal symmetry because $H(x,\theta) = \sigma_y H^*(x,\theta)\sigma_y$. Note that both these symmetries map a current, I, along the wire such that $I \to -I$ and as such if either symmetry is present it requires that the resistance $R(I) = R(-I)$ and no MCA can occur. As will be discussed below a non-uniform potential $\delta\mu(\theta) \neq \delta\mu(\theta+\pi)$ through the cross-section breaks inversion symmetry and time reversal symmetry can be broken via the application of an external magnetic field.

Supplementary Note 2. Band structure and influence of gating

Applying the spinor rotation $U(\theta) = e^{i\theta\sigma_x/2}$ and using the ansatz wavefunction $\psi_{k\ell\tau}(y,\theta) = \chi_{k\ell\tau}e^{i(\ell\theta+ky)}$ gives

$$U^\dagger HU = v_F\sigma_x\ell/R - v_F k\,(\sigma_y\cos\varphi + \sigma_z\sin\varphi)\,, \quad (2)$$

here k is the momentum along the nanowire and ℓ the angular momentum around the nanowire. Due to the 2π anti-periodicity of $U(\theta)$ the quantum confined surface states of a TI nanowire are such that the states have half-integer angular momentum $\ell = \pm\frac{1}{2}, \frac{3}{2}, \ldots$ around the nanowire and obey the dispersion relation

$$\epsilon_\ell(k) = \pm v_F \sqrt{k^2 + (\ell/r)^2}. \tag{3}$$

Application of a gate voltage, for instance, from the top of the TI nanowire, induces a non-uniform chemical potential $\mu(\phi) = \mu + \delta\mu(\phi)$ in the nanowire cross-section [3–5], where μ is the chemical potential on the nanowire surface (measured from the Dirac point) and $\phi = 0$ is the angle from the direction normal to the gate. The non-uniformity of chemical potential breaks inversion symmetry and can result in a large spin-splitting of the subbands for finite momentum k along the nanowire (see Fig. 1c main text). For an idealised circular nanowire and non-uniformities smaller than the subband gap – although also valid for larger non-uniformities – it has been shown that the dispersion is given by (see Ref. 5 for details)

$$\varepsilon_\ell^\pm(k) \approx \epsilon_\ell(k) \pm \frac{k\mu_{2\ell}}{\sqrt{k^2 + (\ell/r)^2}}, \tag{4}$$

and similarly $-\varepsilon_\ell^\pm(k)$ below the Dirac point. Here the bands are labelled by $\ell > 0$ and μ_n is the nth Fourier coefficient of the non-uniform chemical potential $\delta\mu(\phi) = \sum_n \mu_n \cos n\phi$ assuming the potential is symmetric for $\pm\phi$. The magnitude of the splitting does not depend strongly on the exact shape of the potential, $\delta\mu(\phi)$, other than through the components μ_n [5]. The band splitting due to such a non-uniform potential is shown in Fig. 1c in the main text.

When inversion symmetry in the Hamiltonian Eq. (2) is broken by a non-uniform chemical potential the states in the split-bands possess a finite spin polarisation $\boldsymbol{s} = (0, s_y, s_z)$ in the yz-plane that is perpendicular to the wire axis [5]. As a result, as discussed in the main text, when a magnetic field is applied in the yz-plane the bands shift relative to each other by the Zeeman energy $\pm(g_y\mu_B s_y B_y + g_z\mu_B s_z B_z)/2$ with $g_{y(z)}$ the g-factor in the $y(z)$-direction and μ_B is the Bohr magneton. Given that in $(Bi_{1-x}Sb_x)_2Te_3$ the g-factor is highly anisotropic, $g_z/g_y \simeq 20$ [1, 6], we neglect the contribution $g_y\mu_B s_y B_y$ which would be only significant for very large s_y/s_z. As such, the situation can be modelled analytically by considering helical bands – valid in the limit of small non-uniformities – such that the subbands $\varepsilon_\ell^\pm(k)$ above the Dirac point are given by

$$\varepsilon_\ell^\pm(k) \approx \epsilon_\ell(k) \pm \frac{k\mu_{2\ell}}{\sqrt{k^2 + (\ell/r)^2}} \pm g_z\mu_B s_z B_z/2 \tag{5}$$

and similarly $-\varepsilon^{\pm}_{\ell}(k)$ below the Dirac point (see Fig. 1c main text).

Supplementary Note 3. Influence of orbital effects

We can estimate the field strength at which orbital effects due to the out-of-plane field affect the MCA by considering the probability density of a state in a Landau level without any confinement. Such a state is localised with the form $|\psi|^2 \propto e^{-r^2/2l_B^2}$, where r the displacement on the surface and $l_B = \sqrt{\hbar/eB}$ the magnetic length. As such a Landau level state will only be consistent with the confinement of our $W \approx 200$ nm wide nanowire when $(W/2)/2l_B \sim 1$, this corresponds to a field strength of $B \sim 0.15$ T. Indeed, experimentally we find that at this field strength the MCA begins to saturate (see e.g. Fig. 2b main text).

Orbital effects due to a magnetic field B_x along the wire should also open a gap in the dispersion [5]. However, for the small $B = 110$ mT field used in the experimental angular rotation α the size of such a gap will be very small – since this field strength is considerably less than a flux quantum corresponding to a field value $B \sim 1.4$ T for the given nanowire cross-section – as such orbitals effects will only alter the MCA from the perpendicular component for a very small range of chemical potentials and only for $\alpha \sim 90^{\text{o}}$ or $\alpha \sim 270^{\text{o}}$ in Fig. 2d of the experiment. Finally we note that for a magnetic field, B_x, parallel to the wire axis, the Zeeman contribution can be entirely absorbed into the orbital contribution [5], such that $\frac{\Phi}{\Phi_0} \to \frac{\Phi}{\Phi_0} - \frac{\frac{1}{2}g_x\mu_B B_x}{\hbar v_F/r}$, where $\Phi = BA$ with A the cross-sectional area of the wire, the fundamental flux quantum $\Phi_0 = h/e$, and $\frac{1}{2}g_x\mu_B B_x$ is the Zeeman energy due to the magnetic field parallel to the wire.

Supplementary Note 4. Linear and second order response conductivity from the Boltzmann equation

Since the gate and magnetic field break inversion and time-reversal symmetry, respectively, an MCA is symmetry allowed for the setup shown in Fig. 1b. In this section, we use the Boltzmann equation in the relaxation time approximation to derive the conductivity expanded to linear and second order in electric field, i.e. the linear and second order conductivity [7, 8]. This approach is valid in the diffusive limit, relevant for the bulk-insulating TI nanowires in our experiment [4]. We start by considering a scattering time which is independent of the chemical potential $\tau(\mu) = \tau$. Later in this section we will consider the influence of broadening processes such as Coulomb disorder and the inclusion of the dependence of scattering time on chemical potential.

Boltzmann equation for 1st order conductivity: We begin by expanding the distribution function of states in a given subband labelled by $(\ell, \pm)$ as $f^{\pm,\ell} = f_0^{\pm,\ell} + f_1^{\pm,\ell} + f_2^{\pm,\ell} + ...$, where $f_n^{\pm,\ell}$ is the nth order response to an electric field E of the $\pm$ branch of ℓth subband [7]. The distribution function to first order in E within the relaxation time approximation of the Boltzmann equation is therefore given by

$$f_1^{\pm,\ell} \approx \frac{e\tau E}{\hbar}\frac{\partial f_0^{\pm,\ell}}{\partial k} = e\tau E v_\ell^{\pm}(k)\frac{\partial f_0^{\pm,\ell}}{\partial \varepsilon_\ell^{\pm}(k)}, \tag{6}$$

where $v_\ell^{\pm}(k) = \frac{1}{\hbar}\partial_k \varepsilon_\ell^{\pm}(k)$ is the velocity and e the elementary charge. We use the Fermi-Dirac distribution function for $f_0^{\pm,\ell} = f_0(\varepsilon_\ell^{\pm}(k) - \mu)$.

Next, we calculate the current density $j = j^{(1)} + j^{(2)} + \dots$ expanded order by order in electric field. We begin by utilising the first order distribution function to calculate the current density $j^{(1)}$ which is first order in electric field E (with coefficient given by the linear response conductivity $\sigma^{(1)}$) such that

$$\begin{aligned} j^{(1)} \equiv E\sigma^{(1)} &= -e\sum_{\eta=\pm}\sum_{\ell=\frac{1}{2},\frac{3}{2},\dots}\int \frac{dk}{2\pi} v_\ell^{\eta}(k) f_1^{\eta,\ell} \\ &= e^2 E\sum_{\eta=\pm}\sum_{\ell=\frac{1}{2},\frac{3}{2},\dots}\tau\int \frac{dk}{2\pi}[v_\ell^{\eta}(k)]^2\delta(\mu - \varepsilon_\ell^{\eta}(k)) \\ &= \frac{e^2}{h}E\sum_{\eta=\pm}\sum_{\ell=\frac{1}{2},\frac{3}{2},\dots}\tau\left[v_\ell^{\eta}(k_{\ell,R}^{\eta}) - v_\ell^{\eta}(k_{\ell,L}^{\eta})\right], \end{aligned} \tag{7}$$

where in the second line we used that the Fermi-Dirac distribution at zero temperature is given by $\frac{\partial f_0^{\pm,\ell}}{\partial \varepsilon_\ell^{\pm}(k)} = -\delta(\mu - \varepsilon_\ell^{\pm}(k))$ and, as in the main text, $k_{\ell,R}^{\eta}$ and $k_{\ell,L}^{\eta}$ are right and left Fermi wave vectors for the $\eta = \pm$ branch of ℓth subband (see Fig. 1c main text). In general, these wave vectors $k_{\ell,L/R}^{\eta}$ can be found by numerically solving the equation $\varepsilon_\ell^{\eta}(k_{\ell,L/R}^{\eta}) = \mu$. Note that if the band is not occupied then it will not contribute to the current density j. Finally, we also note that setting magnetic field to zero ($B = 0$) and non-uniform chemical potential to zero ($\delta\mu(\phi) = 0$) we find that $\sigma^{(1)}(\mu)$ coincides with the result with the same limit in Ref. [4] derived from the Kubo formula.

Boltzmann equation for 2nd order conductivity: The E^2 contribution to the current density $j^{(2)}$ can be obtained similarly by using the first order of the distribution function $f_1^{\pm,\ell}$ from above

such that in the relaxation time approximation [7] we arrive at

$$f_2^{\pm,\ell} \approx \frac{e\tau E}{\hbar}\frac{\partial f_1^{\pm,\ell}}{\partial k} = \left(\frac{e\tau E}{\hbar}\right)^2 \frac{\partial^2 f_0^{\pm,\ell}}{\partial k^2}. \tag{8}$$

Inserting this expression for $f_2^{\pm,\ell}$ into the Boltzmann equation gives the component of current density proportional to the square of the electric field as

$$\begin{aligned} j^{(2)} \equiv \sigma^{(2)}E^2 &= -e\sum_{\eta=\pm}\sum_{\ell=\frac{1}{2},\frac{3}{2},\ldots}\int\frac{dk}{2\pi}v_\ell^\eta(k)f_2^{\eta,\ell} = -e\left(\frac{e\tau E}{\hbar}\right)^2\sum_{\eta=\pm}\sum_{\ell=\frac{1}{2},\frac{3}{2},\ldots}\int\frac{dk}{2\pi}v_\ell^\eta(k)\frac{\partial^2 f_0^{\pm,\ell}}{\partial k^2} \\ &= -e\left(\frac{e\tau E}{\hbar}\right)^2\sum_{\eta=\pm}\sum_{\ell=\frac{1}{2},\frac{3}{2},\ldots}\int\frac{dk}{2\pi}v_\ell^\eta(k)\frac{\partial^2\varepsilon_k^\eta}{\partial k^2}\delta(\mu-\varepsilon_k^\eta) \\ &= -\frac{e^3}{h}E^2\sum_{\eta=\pm}\sum_{\ell=\frac{1}{2},\frac{3}{2},\ldots}\tau^2\left[\mathcal{V}_\ell^\eta(k_{\ell,R}^\eta)-\mathcal{V}_\ell^\eta(k_{\ell,L}^\eta)\right], \end{aligned} \tag{9}$$

where $\mathcal{V}_\ell^\pm(k) = \frac{1}{\hbar^2}\partial_k^2\varepsilon_\ell^\pm(k)$ denotes the band curvature and the second line requires integration by parts. We use this to aquire the MCA rectification coefficient

$$\gamma = \frac{-\sigma^{(2)}}{B(\sigma^{(1)})^2} = \sum_{\ell=\frac{1}{2},\frac{3}{2},\ldots}\gamma_\ell, \tag{10}$$

with γ_ℓ given by Eq. (1) of the main text. We note that for a purely parabolic band, for example, in a Rashba nanowire where to good approximation $\varepsilon_\pm(k) = (k \pm k_{\rm so})^2/2m$, the quantity $\mathcal{V}_\ell^\pm(k) = 1/m$ is independent of the Fermi wavevectors $k_{L/R}^\pm$ if the magnetic field points along the spin-orbit interaction vector and, thus, the two contributions $\gamma_\ell^\pm$ of Eq. (1) in the main text would cancel each other. Hence, the giant size of the MCA is a unique feature of the peculiar dispersion of TI subbands and the fact the spin degree of freedom enters Dirac Hamiltonian of the TI surface states. In what follows we focus on the contribution of an individual subband pair via γ_ℓ. We note that the precise magnitude of the harmonics μ_n depends on several experimental factors which are difficult to model theoretically, these include the electrostatics of the nanowire setup, screening effects (e.g. from contacts), and the exact geometry of the nanowires. Using Eq. (9) and the eigenenergies from Eq. (5) gives the result shown in Fig. 1d of the main text. Finally, we note that $\sigma^{(2)}$ is zero when the chemical potential is uniform in the cross-section (i.e. $\mu_n = 0$ for all n) or when the magnetic filed is absent (i.e. $g\mu_B B_y/2 = 0$). In other words there is only a finite MCA when *both* inversion symmetry and time reversal symmetry are broken simultaneously, as expected.

Supplementary Note 5. Estimate of rectification effect:

To obtain a theoretical estimate of the size of the maximum possible MCA rectification γ in a typical nanowire, we first use the experimental fact that the Ohmic resistance of a $L \sim 5$ μm nanowire (e.g. the resistance of Device 3, Section 5, see Fig. S11a) saturates at $R \sim 60$ kΩ as function of gate voltage with only small fluctuations around this value, these oscillation can be attributed to the quantum confinement of surface states (see below). For the $W \approx 200$ nm and $H \approx 16$ nm nanowires used in our experiment the presence of these fluctuations suggests that the broadening due to disorder is smaller than the subband spacing, i.e. $\tau \gtrsim \hbar/(1\ \text{meV}) \sim 1$ ps, in fact this value also fits well with the $\sigma^{(1)} \sim 0.1\ \mu\text{m/k}\Omega$ found experimentally. Numerically calculating $\sigma^{(2)}(\mu)$ using Eq. (9) we find that the maximum $|\sigma^{(2)}| \sim e^3(\tau/\hbar)^2(2500\ \text{meV nm}^2)/h$ close to the bottom of the subband pair, here we use the small angular momenta $\ell = 3/2$, Fourier coefficient $\mu_{2\ell} \sim \ell v/r$, $v \sim 5 \times 10^5$ m/s, and $B \sim 0.05$ T with the g-factor $g_z \approx 50$ [1] and take a conservative estimate for spin component in the z-direction $s_z = 0.08$. Therefore, we estimate the order of magnitude for the maximal rectification γ_ℓ from a given subband pair as

$$|\gamma_\ell^{\max}| = \frac{|\sigma^{(2)}|}{B(\sigma^{(1)})^2} \sim \frac{e^3(1\ \text{ps}/\hbar)^2(2500\ \text{meV nm}^2)}{h(0.05\ \text{T})(0.1\ \mu\text{m/k}\Omega)^2} \sim 5 \times 10^5\ \text{A}^{-1}\text{T}^{-1}. \tag{11}$$

This estimate represents only an approximation of the order of magnitude that one can expect due to MCA in TI nanowires, showing that it is extremely large. In real experimental systems the value of $|\gamma_\ell^{\max}|$ could even be larger if the non-uniformity of chemical potential across the nanowire cross-section, characterised by the Fourier harmonics μ_n, is larger than assumed here. It is, however, as found in experiment, more likely that $|\gamma_\ell^{\max}|$ is reduced in size by several factors such as the chemical potential dependence of scattering time, smearing of chemical potential due to Coulomb disorder, and further effects not considered in this simple approximation, for instance the separate contributions of bulk and surface conductivity channels. Note that throughout the calculations above, there is an assumption that $|E\sigma^{(2)}| \ll |\sigma^{(1)}|$ is always satisfied; when this assumption is not satisfied, our second-order theory breaks down. Although the rectification is giant in comparison to the largest γ values observed so far, the estimate given in Eq. (11) is still orders of magnitude below this bound for all values of B and I in our experiment.

Supplementary Note 6. Influence of disorder

Influence of Coulomb disorder: One reasons for the lower experimental value than the estimate of Eq. (11) is the influence of Coulomb disorder in the bulk and on the surface of the TI nanowire [9–12]. In general, such Coulomb disorder leads to fluctuations in the local chemical potential of the nanowire surface, resulting in so-called surface puddles. These surface puddles smear out the chemical potential and lead to an averaging of γ_ℓ over the length of the nanowire [12]. To approximate the influence of such surface puddles we use our result from Eq. (9) and assume that the surface fluctuations of chemical potential belong to a Gaussian distribution of width Γ, such that

$$\bar{\gamma}_\ell(\mu) = \frac{1}{\Gamma\sqrt{2\pi}} \int_{-\infty}^{\infty} d\mu' \gamma_\ell(\mu') e^{-\frac{1}{2}\left(\frac{\mu-\mu'}{\Gamma}\right)^2}. \quad (12)$$

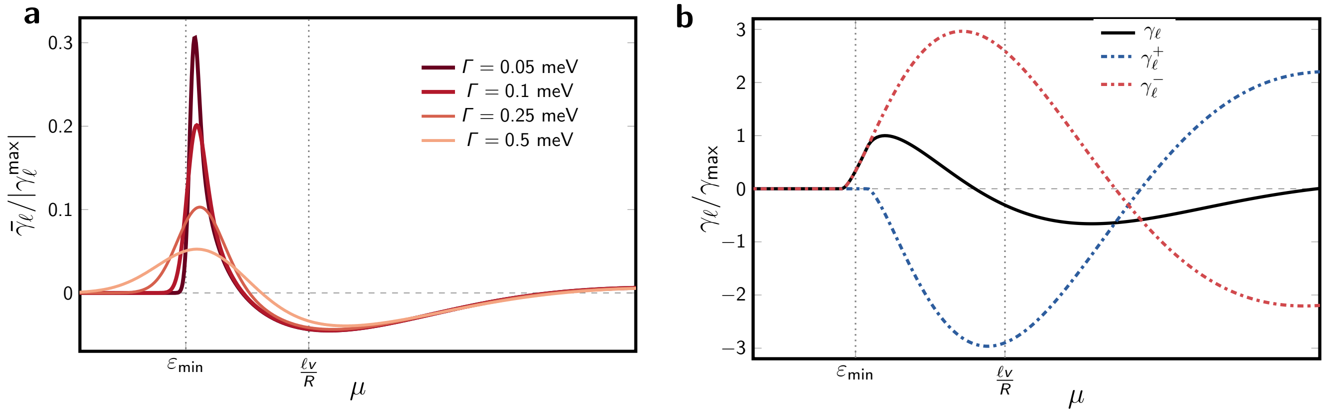

FIG. S1: **Influence of different disorder effects: a,** Influence of Coulomb disorder: Charged impurities in the bulk and on the surface of the TI smear out the chemical potential μ which leads to a reduction and smoothing of γ_ℓ, here shown for a constant scattering time τ. We choose realistic experimental parameters $B = 0.05$ T, $g = 4$, $v_F = 5 \times 10^5$ m/s, $\tau \sim 1$ ps. For this plot we also chose $\ell = 3/2$ with the Fourier harmonic $\mu_{2\ell} \approx v_F\ell/r \approx 3.9$ meV. This gives $|\gamma_\ell^{\max}| \sim 5 \times 10^5$ $\mathrm{T^{-1}A^{-1}}$ as discussed in the text. These are the same parameters as in Fig. 1d of the main text, apart from there $B = 1$ T is used for clarity of the different contributions. **b,** Influence of strong subband minimum scattering: Although only weakly visible in our experimental devices, close to a given subband minimum there is an increase in the scattering rate $1/\tau$ due to the diverging density of states [4]. Here, we show that, even in nanowires in which τ is very strongly dependent on chemical potential μ (such that $\tau = 0$ at the bottom of the band), the overall qualitative features of γ_ℓ will be largely unaffected by such a divergence, with a pronounced maximum and change in sign still clearly visible. We use the same parameters as in **a**, apart from $B = 1$ T for clarity of the different contributions. For simplicity in this plot we assume $\sigma^{(1)} \approx$ const. since there are more conductivity channels for Ohmic conductivity, i.e. through the bulk. In reality $\sigma^{(1)}$ will also inherit the additional dependence on τ which will lead to an increase in γ_ℓ close to $\varepsilon_{\min}$.

We show $\bar{\gamma}_\ell(\mu)$ for several distributions widths Γ in Fig. S1. As expected, for not unrealistic broadenings Γ, the smearing results in a smoothening of the function $\gamma_\ell(\mu)$ and a reduction in the maximum possible rectification $|\bar{\gamma}_\ell^{\max}|$. The density of Coulomb disorder and its influence on the surface states of TI nanowires remains an open experimental question, but the presence of quantum confinement oscillations in the resistivity curves suggests that the broadening Γ of our ribbon-shaped experimental nanowires is smaller than or similar to the subband spacing.

Influence of scattering time: In the experiment, quantum confinement features in the Ohmic resistance R due to increased scattering near the bottom of subbands are weak ($\sim 5\%$ of the total R, see Fig. 3a), and so, to a good approximation, τ can be taken as constant (as above). In theory, for a nanowire with perfect quantum confinement and only surface contributions to the conductivity, the scattering time τ becomes very strongly dependent on the position of the chemical potential within a given subband due to the divergence in density of states at subband minima in one dimension [4]. For completeness, here we take the opposite limit and assume the scattering time is strongly dependent on chemical potential and consider how this would affect $\sigma^{(2)}(\mu)$ and hence $\gamma_\ell(\mu)$. We will see that the overall qualitative features of $\gamma_\ell(\mu)$ are largely unchanged in this limit.

To estimate the scattering time we will use the Born approximation [4] valid for weak impurity scattering potential u_0 and low impurity density n_{imp} and assume that spin-flip interband scattering is negligible. The scattering time for the separate $\pm$ bands at zero temperature is given by

$$\frac{\hbar}{\tau^{\pm}(\mu)} \approx n_{\text{imp}} u_0^2 \sum_{\ell=\frac{1}{2},\frac{3}{2},\dots} \int \frac{dk}{2\pi} \delta(\mu - \varepsilon_\ell^{\pm}(k)) = n_{\text{imp}} u_0^2 \sum_{\ell=\frac{1}{2},\frac{3}{2},\dots} \rho_\ell^{\pm}(\mu), \tag{13}$$

where

$$\rho^{\pm}(\mu) = \sum_{\ell=\frac{1}{2},\frac{3}{2},\dots} \left(\frac{1}{\hbar |v_\ell^{\pm}(k_{\ell,L}^{\pm})|} + \frac{1}{\hbar |v_\ell^{\pm}(k_{\ell,R}^{\pm})|} \right) \tag{14}$$

is the density of states of the respective $\pm$ bands. The linear response and second harmonic conductivities are obtained with the replacement $\tau \to \tau^{\pm}(\mu)$. The density of states in Eq. (14) diverges at the bottom of a given subband leading to a rapid increase in the scattering rate at this point [4], however, the qualitative features of $\sigma^{(2)}(\mu)$ and hence γ_ℓ within a given subband are not strongly modified by this additional dependence of $\tau(\mu)$ (see Fig. S1b). Additionally, the divergence of scattering rate will be cut-off by temperature and disorder, we can therefore expect that the true experimental behaviour of γ_ℓ is somewhere between the two scenarios shown

in Fig. S1b and Fig. 1d of the main text.

EXPERIMENTAL DESCRIPTIONS

Supplementary Note 7. Materials characterization

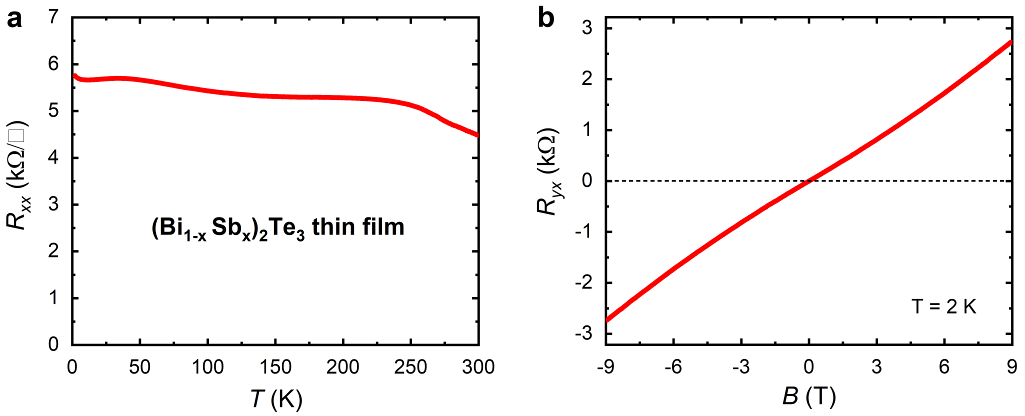

FIG. S2: **Transport properties of the $(Bi_{1-x}Sb_x)_2Te_3$ thin film used for the nanowire device fabrication. a,** Temperature dependence of the sheet resistance R_{xx} of the thin film used in this study. A six-terminal measurement was done on a small part of the 2×2 cm^2 film. **b,** Magnetic-field dependence of the Hall resistance R_{yx} at 2 K.

Figure S2 shows the the temperature dependence of the sheet resistance R_{xx} and the magnetic-field dependence of the Hall resistance R_{yx} measured on a small piece taken from the $(Bi_{1-x}Sb_x)_2Te_3$ (BST) thin film that was used for fabricating all the nanowire devices. The $R_{xx}(T)$ behavior, presenting only a weak T-dependence, is typical for a bulk-insulating BST thin film [13, 14], which is corroborated by the low 2D hole carrier density of only $n \sim 1.8\times10^{12}$ cm^{-2} calculated from dR_{yx}/dB at $B = 0$ T. We also obtain the mobility $\mu \simeq 600$ cm^2/Vs from the data in Fig. S2.
To further document the dominant role of the surface states in the transport properties, Fig. S3 shows the gate-voltage (V_G) dependence of the resistance R_{xx} and the Hall coefficient R_H of Device 4. For these measurements, we reconfigured Device 4, which is on the same wafer as Devices 1–3, to use the nanowire section as the Hall-voltage electrodes (see inset of Fig. S3b), because the Hall voltage cannot be measured on a nanowire; the $R(V_G)$ data were simultaneously measured with a two-terminal configuration on the section where R_H was measured. The V_G-dependence of R_H in Fig. S3b presents the typical semi-divergence and sudden sign change as the Fermi level is tuned across the Dirac point with gating; this behavior gives evidence that the Dirac cone of

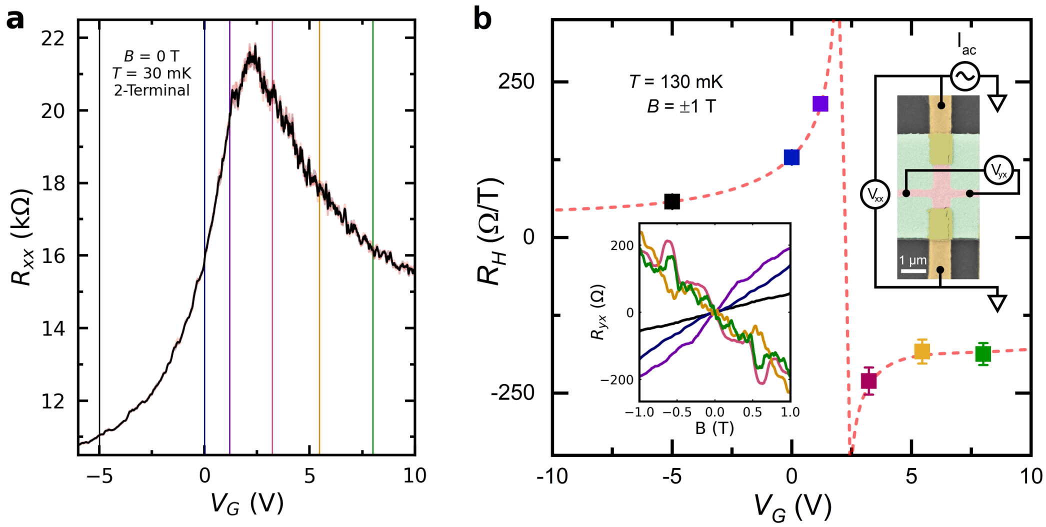

FIG. S3: **V_G-dependence of R_{xx} and R_H in Device 4. a,** $R_{xx}(V_G)$ data measured with a two-terminal configuration shown in the inset of panel **b**. Thin red lines show 3 uni-directional V_G sweeps, while the thick black line shows the average. Coloured vertical lines shows the V_G positions where R_H in panel **b** was measured. **b,** R_H at selected V_G values extracted from a linear fit of the $R_{yx}(B)$ data for $B = \pm 1$ T applied along the $\hat{\boldsymbol{z}}$-axis, which are shown in the left inset. The red dashed line is a guide to the eye. The right inset shows the reconfigured electrode arrangement of Device 4 for these measurements, in which the nanowire part is used as Hall-voltage electrodes.

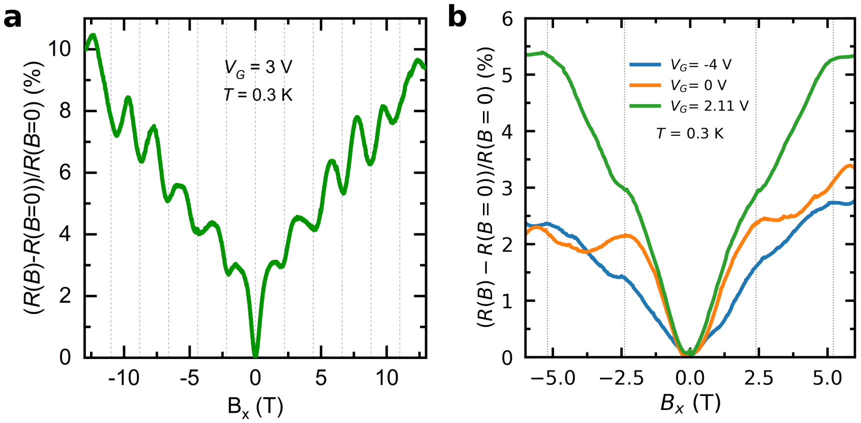

FIG. S4: **Aharonov-Bohm-like oscillations in top-down-fabricated TI nanaowires. a,** Magnetic-field dependence of the resistance, $R(B)$, measured in Device 5, Section 2, with magnetic fields applied along the nanowire axis, normalized by the resistance value at $B = 0$ T. Vertical dashed lines mark the periodicity. **b,** Similar oscillations were observed at various V_G values in Device 4, Section 2, although the maximum magnetic field was limited to 6 T in the measurement of this device. Vertical dashed lines mark the peak positions.

the surface states dominates the transport. In addition, the corresponding V_G-dependence of the resistance R in Fig. S3a presents a sharp peak at the Dirac point, which is another signature of the dominant surface transport.

The magnetoresistance (MR) data with magnetic fields applied along the nanowire axis provide additional support to the dominant role of the surface states when they present Aharonov-Bohm (AB)-like oscillations (see Ref. 15 for details) which reflect the periodic change in the quantum-confined surface states as a function of the threading magnetic flux [16]. The MR data shown in Fig. S4a were measured on Device 5, which was fabricated in the same way and from a thin film of very similar quality as that of Devices 1–4. The nanowire part of Device 5 was ∼17-nm thick and ∼200-nm wide. The observed oscillation period of ∼2.2 T corresponds to the flux quantum Φ_0 ($= h/e$) if the relevant area is 1.9×10^{-15} m^2, which is essentially consistent with the cross-sectional area of the nanowire when considering the finite extension of the surface-state wavefunction. This consistency gives confidence in the interpretation that the MR data in Fig. S4a signifies the AB-like oscillations from the quantum-confined surface states. The TI nanowire of Divice 4 used for the MCA experiment presents similar AB-like oscillations at various V_G values as shown in Fig. S4b, although the magnetic-field range was limited to ±6 T in the measurement of this device. Here again, the oscillation period of ∼2.6 T (indicating the relevant area of 1.6×10^{-15} m^2) is essentially consistent with the cross-sectional area of the nanowire ($\sim 16 \times 200$ nm^2) considering the depth of the surface states.

Supplementary Note 8. Signatures of well-defined surface state subbands

The regular gate dependent resistance oscillations and AB-like resistance oscillations in magnetic field that we observe are both signatures of well-defined subbands [3, 4, 16–18]. Both types of oscillation are of similar magnitude, this is expected since they arise as the result of the increased density of states that occurs close to the bottom of subbands [4]. In this section we will show that both gate dependent resistance oscillations and AB-like oscillation depend on the nanowire cross-sectional geometry and that the estimate of this geometry is consistent with the geometry of our nanowires for both types of oscillation. We also note that the TI nanowire devices used in our study have long channel lengths up to several μm, we can therefore confidentially rule out the presence of universal conductance fluctuations (UCFs) in our TI nanowire devices since the channel lengths are longer than the phase coherence length and, in addition, we do not observe the magnetic field dependent resistance jumps expected to arise due to UCFs (see Fig. S4). Finally, the observation of the smooth change of sign of the MCA signal within single gate voltage peaks actually provides yet another check for well defined subbands in our nanowires.

The nanowires in our study have widths of $W \approx 200 \pm 20$ nm and heights $H \approx 16 \pm 1$ nm, this

leads to variations between different devices of ±15% in cross-sectional area $A = W \times H$ and variations ±10% in the perimeter $P = 2(W + H)$. As discussed above, the observed AB period, $P_{\rm AB}$, was ∼2.6 T for Device 4 (16 nm thickness measured via AFM) and ∼2.2 T in Device 5 (17 nm measured via AFM). The periods of AB-like resistance oscillations are dependent on the flux enclosed by the quantum confined surface state and as such vary with the cross-sectional area, A, of the device. Therefore, taking into account the ∼ 4 nm average extension of the TI surface states [19], the periods of AB-like oscillations allow us to estimate a width $W \approx 190$ nm for Device 4 and $W \approx 220$ nm for Device 5. These values fall within the expected range of the nanowire widths.

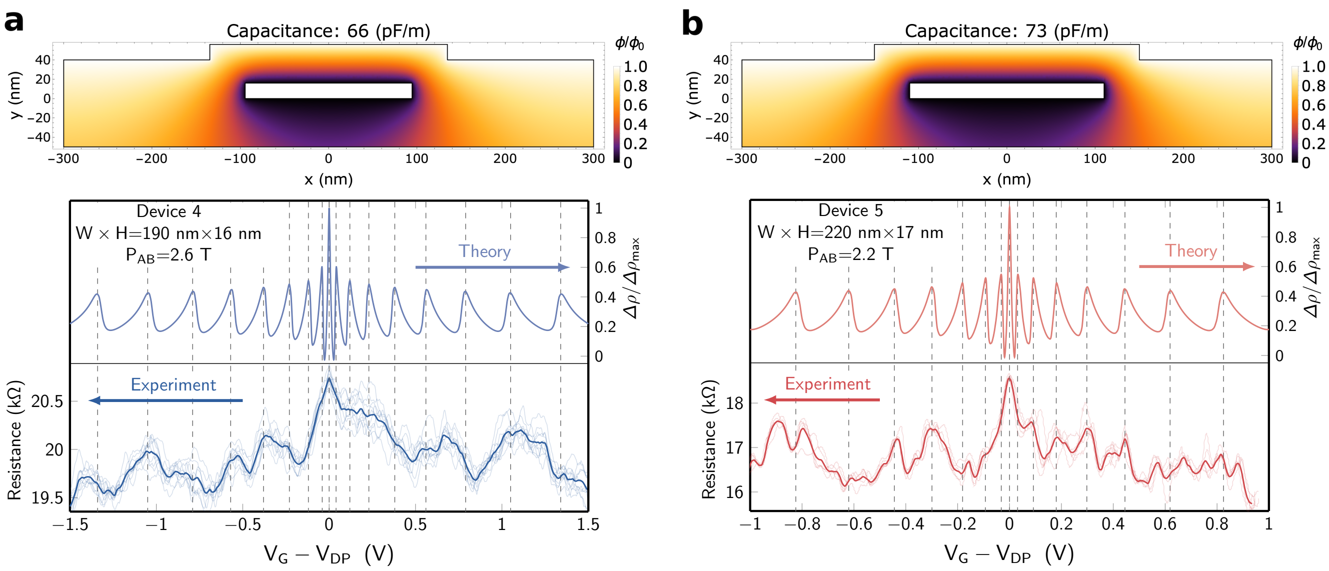

FIG. S5: **Gate voltage dependent resistance oscillations close to the resistance maximum:** a) Device 4 and b) Device 5. Top: Electrostatic potential resulting from numerical solution of the Laplace equation using an electrostatic model of our full device setup and the device cross-section estimated from AB-like oscillations. Bottom: Gate voltage dependent resistance averaged over several gate voltage sweeps (lighter thin lines show individual gate sweeps). The theoretically predicted locations of peaks (dashed lines), which are calculated using the electrostatic model and dimensions of the nanowire cross-section, coincide relatively well with the observed resistance curves, especially for small subband indices ℓ. This confirms that the spacing of gate dependent resistance peaks is related to the cross-sectional geometry. The resistance maximum occurs at $V_{\rm DP} = 4.01$ V for Device 4 and $V_{\rm DP} = 3.05$ V for Device 5. Model parameters: Al_2O_3 dielectric permitivity $\epsilon = 9.34$, top Al_2O_3 thickness 40 nm, and top Al_2O_3/gate width is nanowire width W plus 40 nm.

Gate voltage dependent resistance oscillations are set by the perimeter, P, and the capacitance of the device, C. Since there are smaller variations in the perimeter than cross-sectional area and it is likely the capacitance also varies between devices, it should be expected that the spacing of gate voltage resistance oscillations contains greater uncertainty compared with the periods of AB-like oscillations. Despite this, following the same analysis as Ref. 4, in Fig. S5 we plot the

resistance $R(V_{\rm G})$ – averaged over several gate sweeps to eliminate artefacts such as e.g. jumps from trapped charges – in the vicinity of the resistance maximum $R(V_{\rm DP})$. We estimate the capacitance of Device 4 and Device 5 by utilising an electrostatic model for our full setup – including dielectric environment – and solving the Laplace equation using the finite element method. We make the simple assumption that the nanowire can be modelled as a perfect metal and that effects of quantum capacitance can be neglected for our setup. This calculation gives a capacitance $C = 66$ pF/m for the parameters of Device 4 and $C = 73$ pF/m for the parameters of Device 5. We use the estimated capacitance and the dimensions of each device to plot the theoretically expected resistance, $R(V_{\rm G})$, and positions of individual gate peaks (dashed lines). Note that the peak position varies with charge density $n \propto \mu^2$, such that the spacing of subband oscillations is approximately quadratic in the voltage difference from the location of the resistance maximum. From Fig. S5, despite the smaller variations in perimeter and additional uncertainty in capacitance, we see that the locations of observed resistance peaks correspond relatively well with the theoretically expected resistance curve, especially for small subband indices ℓ. In Fig. S5 all parameters of the theoretical curves are set by the electrostatic simulations and estimates of the nanowire cross-sectional geometry that are obtained from AB-like oscillations, meaning that there is no free fitting parameter.

Supplementary Note 9. Raw data of $R_{2\omega}$ and symmetric component

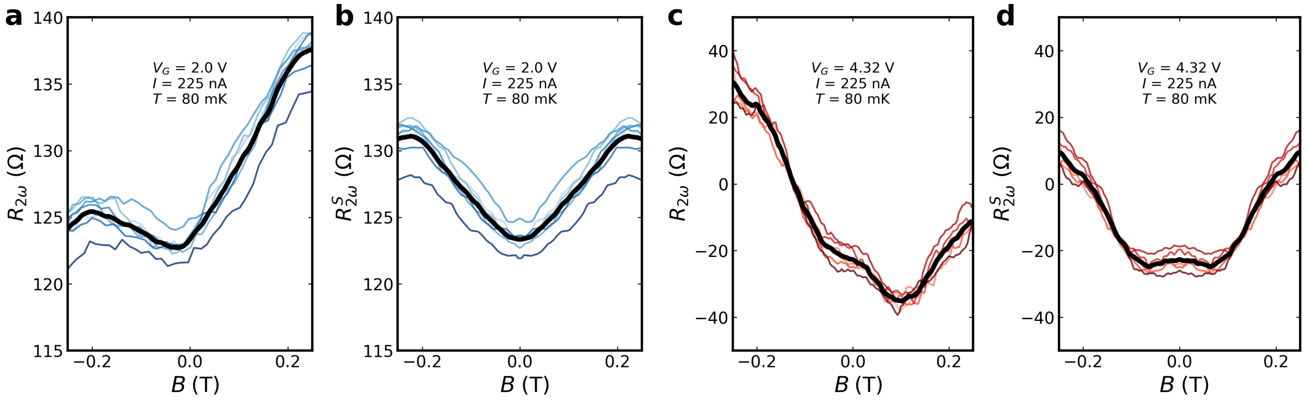

FIG. S6: **Raw $R_{2\omega}$ data and symmetric component $R^S_{2\omega}$ of Device 1, Section 1. a,** Raw $R_{2\omega}$ vs B_z data at $V_G = 2$ V. **b,** The symmetric component $R^S_{2\omega}$ extracted from the raw data for $V_G = 2$ V. **c,** Analogously, raw $R_{2\omega}$ vs B_z data at $V_G = 4.32$ V. **d,** The symmetric component $R^S_{2\omega}$ extracted from the raw data for 4.32 V. The corresponding extracted asymmetric components $R^A_{2\omega}$ are shown in Fig. 2b of the main text. Coloured thin lines show the results of 6 sweeps, and the thick black line shows their average.

Upon sweeping the magnetic field B_z applied parallel to the gate-induced electric field E, the

second-harmonic voltage $R_{2\omega}$ comprises an antisymmetric component $R_{2\omega}^{A}$ and a symmetric component $R_{2\omega}^{S}$; these two components can be easily separated by antisymmetrization/symmetrization of the B-dependence data. Figures S6a and S6c show the raw $R_{2\omega}$ data for $V_G = 2$ V and 4.32 V, respectively, from which the $R_{2\omega}^{A}$ data shown in Fig. 2b of the main text were extracted. The $R_{2\omega}^{S}$ component shown in Figs. S6b and S6d can have various trivial origins such as a diode-like effect at an imperfect contact.

Supplementary Note 10. Magnetic-field-orientation dependence of γ for the in-plane and out-of-plane rotations

In addition to the magnetic-field-orientation dependence of $R_{2\omega}^{A}$ for the zx-plane rotation shown in the main text (Fig. 2d), we have taken similar data on Device 1 for the yz-plane rotation (Fig. S7) and found that the behaviour is also consistent with $\gamma \approx \gamma_0 \cos\alpha_2$, where α_2 is the angle of the B-field away from the z-direction in the yz-plane. Each data point was generated by averaging 6 (8) individual B-field sweeps at $V_G = 2$ V (4.32 V), while the error was calculated by a Min-Max method (though a standard deviation approach gives very similar results). We note that the γ_0 values for the zx- and yz-plane rotations at nominally the same V_G are slightly different; this is probably due to a difference in the details of the charge distributions and a slightly different chemical potentials for the two measurements, since the gating history was different.

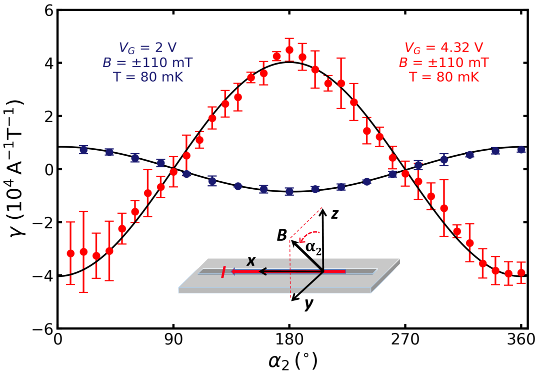

FIG. S7: **Magnetic-field-orientation dependence of γ for the yz-plane rotation in Device 1, Section 1. a,** The MCA coefficient γ obeys $\gamma \approx \gamma_0 \cos\alpha_2$, where α_2 is the angle from the z-direction. The data shown here is for Device 1, Section 1, at V_G of 2 V (blue) and 4.32 V (red). Solid black lines are fits to the cosine function. The inset shows the definition of α_2 and the coordinate system.

We measured the magnetic-field-orientation dependencies for all three major rotation planes (xy, yz, and zx) in Device 4 at $V_G = 3.26$ V and the results are shown in Fig. S8. Each data point was generated by averaging 10 individual B-field sweeps, while the error was calculated by a Min-Max

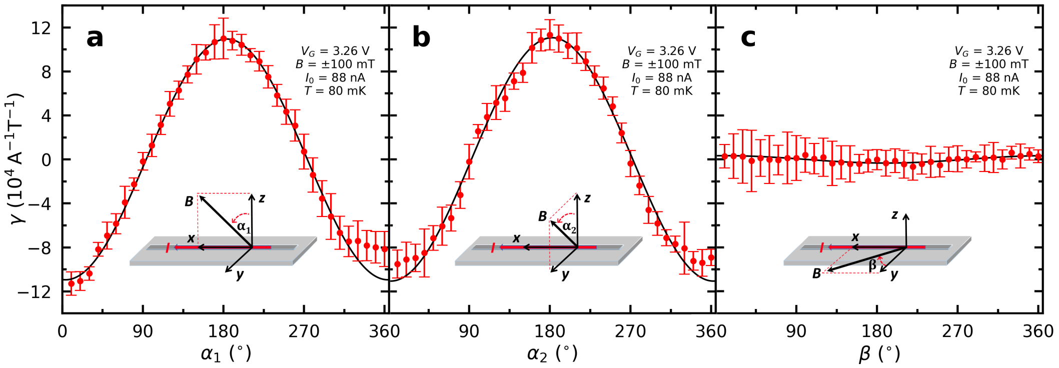

FIG. S8: **Magnetic-field-orientation dependence of γ for Device 4, Section 1. a,b,c** At $V_G = 3.26$ V, the MCA coefficient γ was determined at low fields (-100 mT $\leq B \leq +100$ mT) while the magnetic-field orientation was gradually rotated in the zx, yz, and xy planes, respectively. Solid black lines are fits to the cosine function. Insets show the definitions of α_1, α_2 and β in each rotation plane.

method. One can see that the $\gamma \approx \gamma_0 \cos\alpha$ dependence is reproduced for the zx- and yz-plane rotations, while γ remains essentially zero during the xy-plane rotation, which fully supports the vector-product type MCA with the $\boldsymbol{P}$ vector along the y-axis. Note that the maximum γ value observed in Device 4 reaches 1×10^5 A^{-1}T^{-1} (see Figs. S8a and S8b).

Supplementary Note 11. Reproducibility of the magnetochiral anisotropy

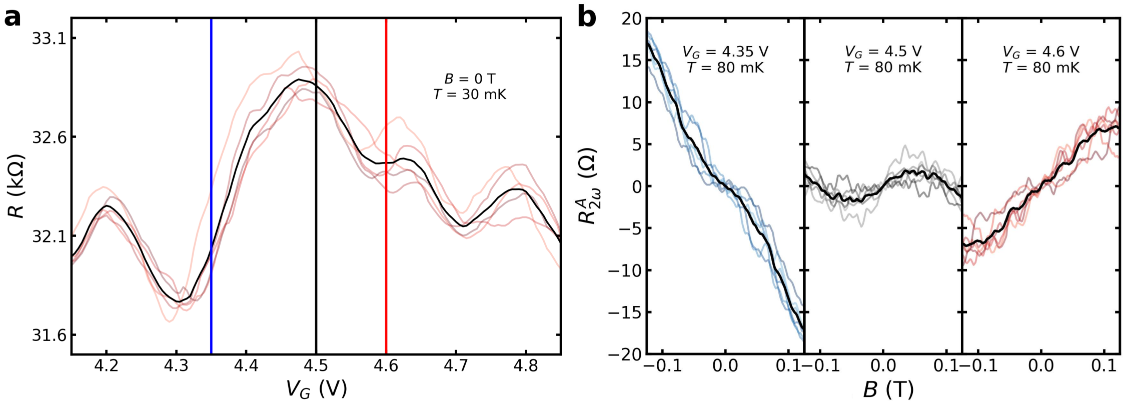

FIG. S9: **Sign change of γ upon changing the chemical potential in Device 1, Section 1. a,** The V_G-dependence of R in the V_G range of 4.15–4.9 V obtained in a different sweep from that in Fig. 2a of the main text. Thin coloured lines are the data from 5 individual V_G sweeps and the thick black line is their average. The blue, black, and red vertical lines mark the V_G values at which the B-dependence of $R^A_{2\omega}$ was measured. **b,** Plots of $R^A_{2\omega}$ vs B_z at the V_G values of 4.35 V, 4.5 V, and 4.6 V, which span a peak in the $R(V_G)$ curve shown in panel **a**. The sign of $dR^A_{2\omega}/dB_z$ near $B_z = 0$ T (i.e. the sign of γ) changes in this V_G interval. Coloured thin lines show 6 individual B-field sweeps and the thick black line shows their average.

To confirm the reproducibility of the experimental data supporting a giant MCA in BST nanowires, additional measurements have been performed on four devices (Device 1, 2, 3, 4) at various nanowire sections. All four devices had the same design (with slightly different width) and were fabricated from the same thin film in the same fabrication batch. The nanowire width was ∼200, ∼205, ∼210, and ∼200 nm in Devices 1 to 4, respectively, with the film thickness of ∼16 nm. In the following, we show representative data reconfirming the behaviours reported in the main text.

Figure S9 shows a data set to confirm the sign change in γ upon a slight change in the chemical potential. The data were taken on Section 1 of Device 1.

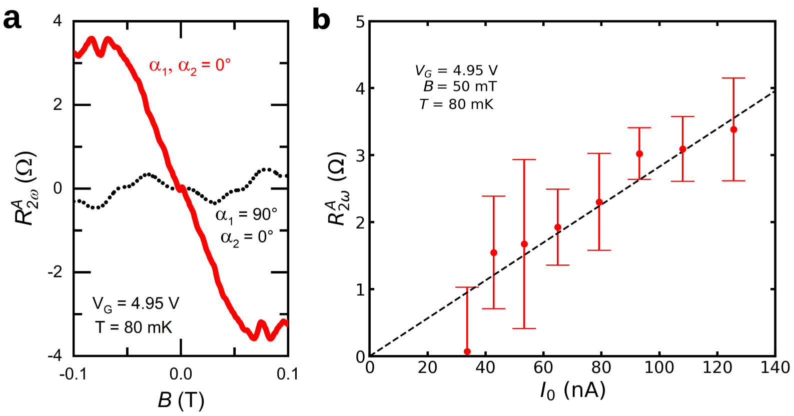

FIG. S10: **MCA in Device 1, Section 2. a,** Averaged $R^A_{2\omega}(B)$ behaviours for $\boldsymbol{B} \parallel \hat{\boldsymbol{z}}$ (red solid line) and $\boldsymbol{B} \parallel \boldsymbol{I}$ (black dotted line) at $V_G = 4.95$ V measured on Section 2 of Device 1. Averages are obtained from 10 individual B-field sweeps. **b,** $R^A_{2\omega}$ at $V_G = 4.95$ V as a function of the ac excitation current I_0 for $B_z = 50$ mT. The black dashed line is a guide to the eye to mark the linear behaviour.

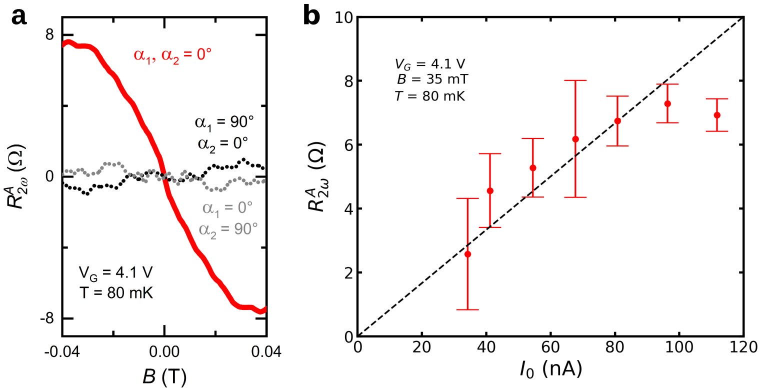

FIG. S11: **MCA in Device 2, Section 3. a,** Averaged $R^A_{2\omega}(B)$ behaviours for $\boldsymbol{B} \parallel \hat{\boldsymbol{z}}$ (red solid line), $\boldsymbol{B} \parallel \boldsymbol{I}$ (black dotted line) and $\boldsymbol{B} \parallel \hat{\boldsymbol{y}}$ (grey dotted line) at $V_G = 4.1$ V measured on Section 3 of Device 2. Averages are obtained from 10 individual B-field sweeps. **b,** $R^A_{2\omega}$ at $V_G = 4.1$ V as a function of the ac excitation current I_0 for $B_z = 35$ mT. The black dashed line is a guide to the eye to mark the linear behaviour.

Figures S10 and S11 show further data, taken on Section 2 of Device 1 and Section 1 of Device 2, respectively, to demonstrate that our BST nanowires consistently show the large MCA. The three basic characteristics of the vector-product-type MCA are: (i) $R^A_{2\omega}$ depends linearly on B at low field, (ii) $R^A_{2\omega}$ is maximum for $\boldsymbol{B} \parallel \hat{\boldsymbol{z}}$ and vanishes when $\boldsymbol{B}$ is along $\hat{\boldsymbol{x}}$ or $\hat{\boldsymbol{y}}$, and (iii) $R^A_{2\omega}$ increases linearly with I. All three characteristics are reproduced.

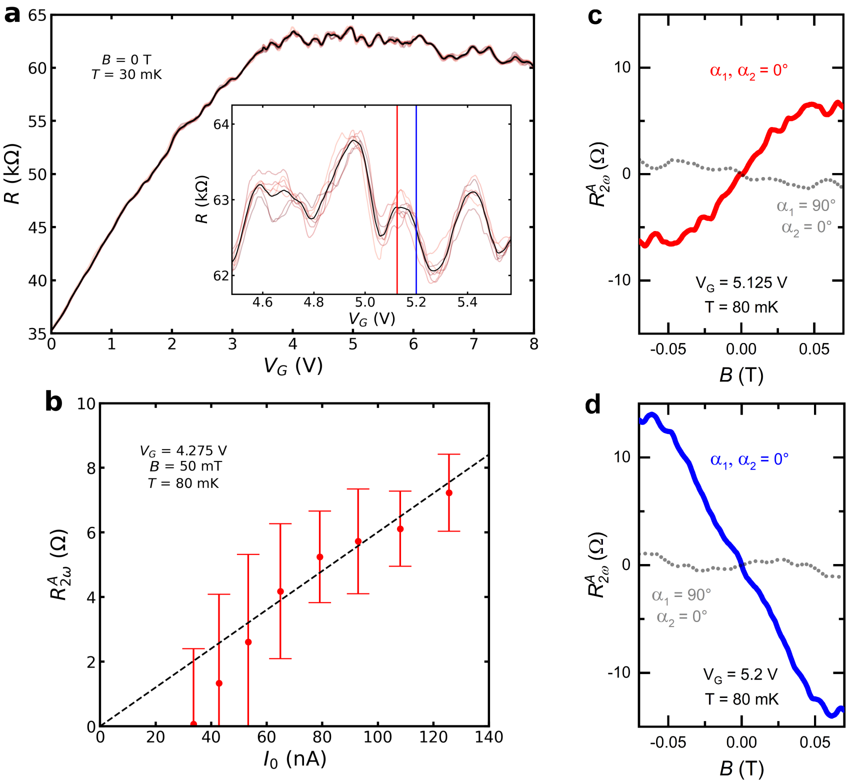

FIG. S12: **MCA in Device 3, Section 5. a,** $R(V_G)$ behaviour of the Section 5 of Device 3 in a wide V_G range, presenting a broad maximum around $V_G \simeq 5$ V. Coloured thin lines show 7 uni-directional V_G sweeps and the thick black line shows the average. Inset shows a magnification of the V_G range 4.5–5.6 V; the red and blue vertical lines mark the V_G positions used for the $R^A_{2\omega}(B)$ measurements shown in panels **c** and **d**. **b,** $R^A_{2\omega}$ at $V_G = 4.275$ V as a function of the ac excitation current amplitude I_0 for $B =$ 50 mT applied along $\hat{\boldsymbol{z}}$. The black dashed line is a guide to the eye. **c,d** Averaged $R^A_{2\omega}(B)$ behaviours for $\boldsymbol{B} \parallel \hat{\boldsymbol{z}}$ (solid lines) and $\boldsymbol{B} \parallel \boldsymbol{I}$ (dotted lines) at $V_G = 5.125$ V (c) and 5.2 V (d). The sign of $dR^A_{2\omega}/dB_z$ near $B = 0$ T (i.e. the sign of γ) changes in this narrow V_G interval crossing a peak in the $R(V_G)$ curve. Averages are obtained from 10 individual B-field sweeps.

Figure S12 shows supplemental data for Device 3 which was used for demonstrating the repeated sign change in γ as a function of V_G in the main text. The V_G dependence of R is shown in Fig. S12a for a wide range of V_G. The I-linear increase of $R^A_{2\omega}$ is shown in Fig. S12b. The B-linear dependence of $R^A_{2\omega}$ for $\boldsymbol{B} \parallel \hat{\boldsymbol{z}}$ and vanishing $R^A_{2\omega}$ for $\boldsymbol{B} \parallel \boldsymbol{I}$ are shown for both positive and negative γ in Figs. S12c and S12d, respectively.

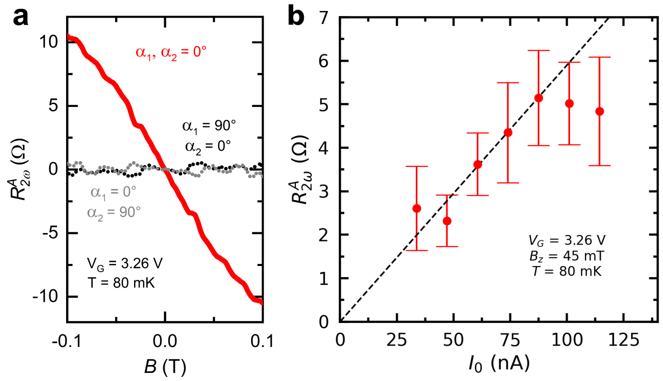

FIG. S13: **MCA in Device 4, Section 1. a,** Averaged $R^A_{2\omega}(B)$ response for $\boldsymbol{B} \parallel \hat{\boldsymbol{z}}$ (red solid line), $\boldsymbol{B} \parallel \hat{\boldsymbol{x}}$ (black dotted line) and $\boldsymbol{B} \parallel \hat{\boldsymbol{y}}$ (grey dotted line) at $V_G = 3.26$ V measured on Section 1 of Device 4. Averages are obtained from 10 individual B-field sweeps. **b,** $R^A_{2\omega}$ at $V_G = 3.26$ V as a function of the ac excitation current I_0 for $B_z = 45$ mT. The black dashed line is a guide to the eye to mark the linear behaviour.

Figure S13 shows that Device 4, which was used for the full angular-dependence measurement (Supplementary Note 10, Fig. S8) and the temperature-dependence measurement (Supplementary Note 12, Fig. S14), also presents giant MCA. The three basic characteristics, B-linear dependence of $R^A_{2\omega}$ for $\boldsymbol{B} \parallel \hat{\boldsymbol{z}}$ at low field, vanishing $R^A_{2\omega}$ when $\boldsymbol{B}$ is along $\boldsymbol{I}$ or $\boldsymbol{P}$, and I-linear dependence of $R^A_{2\omega}$, are all confirmed.

Supplementary Note 12. Temperature dependence of MCA

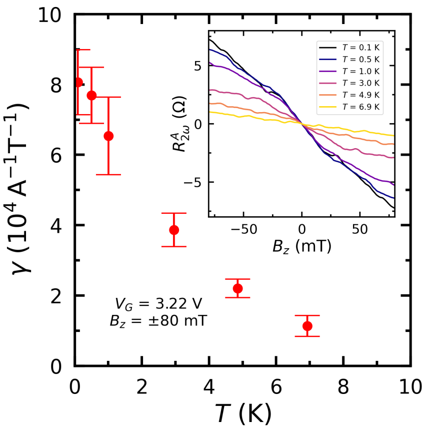

FIG. S14: **Temperature dependence of γ for Device 4, Section 1. a,** The γ value at $V_G = 3.22$ V determined at low fields (-80 mT $\leq B \leq +80$ mT) at various temperatures up to 7 K. Inset shows the raw data of $R^A_{2\omega}$ vs B_z at various temperatures; each curve is an average of 10 individual B-field sweeps.

To elucidate the temperature dependence of MCA, we measured γ of Device 4, Section 1, at V_G = 3.22 V at various temperatures up to 7 K (Fig. S14). We found that MCA weakens with

temperature and diminishes towards ∼10 K. The temperature range in which MCA is observed, up to ∼10 K, is in good correspondence with the expected subband gap of ∼1 meV. It is useful to notice that this energy/temperature scale also dictates the AB-like oscillations in the MR for $\boldsymbol{B} \parallel \hat{\boldsymbol{x}}$, which was reported to diminish above a few Kelvin [18].

Supplementary Note 13. Comparison with MCA in other materials and with other non-reciprocal response

	Material	T (K)	B (T)	ΔR (Ω)	$R_{0,1\omega}$ (Ω)	$\Delta R/R$ (%)	i (A/m^2)	$\Delta R/R/i$ (10^{-8} m^2/A)	I (A)	Area (m^2)	γ (A^{-1}T^{-1})	γ' (m^{2}A^{-1}T^{-1})	MCA type	Ref.
Normal conductors	$(Bi,Sb)_2Te_3$ Device 1 Sec.1	0.08	0.11	36	32150	0.112	5×10^{7}	0.23	1.64×10^{-7}	3.3×10^{-15}	62100	2.1×10^{-10}	Vector-product	This study
	$(Bi,Sb)_2Te_3$ Device 1 Sec.2	0.08	0.05	6	14200	0.043	3.3×10^{7}	0.13	1.1×10^{-7}	3.3×10^{-15}	76800	2.5×10^{-10}	Vector-product	This study
	$(Bi,Sb)_2Te_3$ Device 2 Sec.3	0.08	0.035	14	12250	0.114	3×10^{7}	0.38	1×10^{-7}	3.3×10^{-15}	327000	1.1×10^{-9}	Vector-product	This study
	$(Bi,Sb)_2Te_3$ Device 3 Sec.5	0.08	0.05	14	62750	0.022	3.9×10^{7}	0.06	1.3×10^{-7}	3.3×10^{-15}	34300	1.1×10^{-10}	Vector-product	This study
	$(Bi,Sb)_2Te_3$ Device 4 Sec.1	0.08	0.1	20	22700	0.088	2.7×10^{7}	0.33	8.8×10^{-8}	3.3×10^{-15}	100000	3.3×10^{-10}	Vector-product	This study
	n-type Si FET	300	2	0.8	620	0.13	1×10^{10}	1.3×10^{-3}	1×10^{-3}	1×10^{-13}	0.1	1×10^{-14}	Vector-product	[20]
	[DM-EDT-TTF]$_2ClO_4$	300	3.2	0.59	3000	0.020	2×10^{5}	9.9	1×10^{-3}	5×10^{-9}	0.01	5×10^{-11}	Inner-product	[21]
	Carbon nanotube	2.5	14	23	10000	0.23	3.5×10^{10}	6.6×10^{-4}	3.5×10^{-7}	1×10^{-17}	230	2.3×10^{-15}	Inner-product	[22]
	$SrTiO_3$	7	0.18	0.45	50	0.9	8.3×10^{8}	0.1	5×10^{-4}	6×10^{-13}	20	1.2×10^{-11}	Vector-product	[23]
	BiTeBr	2	9	0.036	1000	0.0037	1.3×10^{9}	2.8×10^{-4}	1.3×10^{-4}	1×10^{-13}	1	3×10^{-12}	Vector-product	[7]
	WTe_2	300	14	0.027	180	0.015	7.1×10^{9}	2.1×10^{-4}	1.2×10^{-3}	1.7×10^{-13}	0.22	3.7×10^{-14}	Vector-product	[24]
	Ge(111)	15	1	120	29000	0.41	1.7×10^{5}	0.41	1×10^{-5}	6×10^{-11}	0.7	4.2×10^{-11}	Vector-product	[25]
	Bi_2Se_3	60	9	0.11	1800	0.0063	1.4×10^{9}	4.6×10^{-4}	5.6×10^{-3}	4×10^{-13}	0.0006	2.4×10^{-16}	Vector-product	[26]
	t-Te	300	1.5	-	-	0.01	1.3×10^{4}	77	6.5×10^{-3}	5×10^{-7}	0.00073	3.6×10^{-9}	Inner-product	[27]
	$ZrTe_5$	3	0.02	0.014	29.6	0.047	8.3×10^{4}	57	2×10^{-4}	2.4×10^{-9}	195	4.7×10^{-7}	Vector-product	[8]
Superconductors	MoS_2	2	0.7	1.8	32	5.7	1.1×10^{9}	0.51	1.7×10^{-5}	1.6×10^{-14}	3800	5.9×10^{-11}	Vector-product	[28]
	WS_2 nanotube	4	2	0.57	40	1.4	4.5×10^{6}	31	3.5×10^{-8}	7.9×10^{-15}	100000	7.9×10^{-10}	Inner-product	[29]
	Bi2Te3/FeTe	6.9	0.5	0.0057	0.36	1.6	6.7×10^{7}	2.4	2×10^{-4}	3×10^{-12}	0.00065	2×10^{-14}	Vector-product	[30]
	InAs 2DEG	1.45	0.09	400	45000	0.89	-	-	2.8×10^{-8}	-	4.15×10^{6}	-	Vector-product	[31]

TABLE S1: **Magnetochiral anisotropy in normal conductors and superconductors:** Adapted from Ref. [32] and some new entries, including all our samples, are added. When the values of the MCA coefficient γ and/or γ' were not given in the publication, they are calculated.

	Material	T	B	ΔR	$R_{0,1\omega}$	$\Delta R/R$	i	$\Delta R/R/i$	I	Area	Ref.
		(K)	(T)	(Ω)	(Ω)	(%)	(A/m^2)	(10^{-8} m^2/A)	(A)	(m^2)	
Magnet/ SC	YIG/MoGe	4	7.3	0.073	0.86	8.5	5.7×10^{4}	14900	0.00004	-	[33]
Magnet	Pt/Co	300	2	0.2	290	0.069	4.9×10^{11}	1.4×10^{-4}	0.0213	4.3×10^{-14}	[34]
	Ta/Co	300	0.2	0.014	574	0.0025	1.0×10^{11}	2.5×10^{-5}	0.0085	8.5×10^{-14}	[35]
	Pt/Py	300	0.015	0.04	40	0.1	1.1×10^{12}	9.1×10^{-5}	0.0033	3.0×10^{-15}	[36]
	Pt/Co	300	0.2	0.0031	170	0.0018	1.0×10^{11}	1.8×10^{-5}	0.003	3.0×10^{-14}	[37]
	Co_2MnSi/Pt	300	0.075	0.05	33000	0.00015	1.7×10^{11}	8.9×10^{-7}	0.41	2.4×10^{-11}	[38]
	GaMnAs	130	0.002	2.2	1720	0.13	7.5×10^{9}	0.0017	0.00015	2.0×10^{-14}	[39]
	MnSi	35	0.4	0.00011	1	0.011	1.0×10^{9}	0.0011	0.0025	2.5×10^{-12}	[40]
	$(Bi,Sb)_2Te_3$/ CoFeB	150	0.02	0.0085	730	0.0012	6.7×10^{9}	1.7×10^{-4}	0.0020	3.0×10^{-13}	[41]
	$(Bi,Sb)_2Te_3$/ $Cr_x(Bi,Sb)_{2-x}Te_3$	2	0.7	35	14000	0.25	6.3×10^{6}	3.97	5.0×10^{-7}	8.0×10^{-14}	[42]
	$(Bi,Sb)_2Te_3$/ $Cr_x(Bi,Sb)_{2-x}Te_3$	1.9	1	51	14000	0.23	2.5×10^{6}	15	6×10^{-7}	2.4×10^{-13}	[43]
	$CrNb_3S_6$	<200	2	$\mathcal{O}(10^{-6})$	-	-	1.1×10^{9}	-	0.0045	4.1×10^{-12}	[44]
QAHI	$Cr_x(Bi,Sb)_{2-x}Te_3$	2	0.1	4100	16000	26	1.3×10^{8}	21	0.0001	8.0×10^{-13}	[32]
	$Cr_x(Bi,Sb)_{2-x}Te_3$	0.5	0.1	190	16000	1.1	3.8×10^{6}	31	3×10^{-6}	8.0×10^{-13}	[32]

TABLE S2: **Non-reciprocal responses in magnetic materials/structures:** Adapted from Ref. [32] and some new entries are added. Here, the non-reciprocal responses are possible due to spontaneously broken time-reversal symmetry. Hence, the magnetic field B is playing only a supportive role and γ is not a well-defined parameter to characterize the non-reciprocal response. SC and QAHI stand for superconductor and quantum anomalous Hall insulator, respectively.

To put the giant MCA we observed in gated TI nanowires into context, we compare it with the MCA observed in other materials. Table S1 lists the materials for which relatively large MCA has been reported. In this table, the values of both γ and γ' ($= \gamma A$, with A the cross-sectional area) are given for each material/structure, even though γ' is not an appropriate parameter to measure the MCA rectification in a mesoscopic system like TI nanowires or carbon nanotubes. Also, we list MCA observed in superconductors in this table, although the physical origin of the non-reciprocal response in superconductors is completely different and it exists only in a very limited range of current and temperature. One can see in this table that the γ value found in this study is the largest ever reported.

In addition to listing the MCA in other materials, we also list the cases of relatively large non-reciprocal responses observed in magnetic materials/structures in Table S2. Since time reversal

symmetry is spontaneously broken in these cases, these non-reciprocal effects do not belong to MCA (in which time reversal symmetry is broken by an applied magnetic field) and hence the MCA rectification coefficient γ is not well-defined. Nevertheless, one can see in this table that the size of the rectification effects, measured with either $\Delta R/R$ or $\Delta R/R/i$ (where i is the current density), can be comparable or even larger than MCA.

---

[1] Liu, C.-X. *et al.* Model hamiltonian for topological insulators. *Phys. Rev. B* **82**, 045122 (2010).

[2] Fu, L. Hexagonal warping effects in the surface states of the topological insulator bi_2te_3. *Phys. Rev. Lett.* **103**, 266801 (2009).

[3] Ziegler, J. *et al.* Probing spin helical surface states in topological HgTe nanowires. *Phy. Rev. B* **97**, 035157 (2018).

[4] Münning, F. *et al.* Quantum confinement of the Dirac surface states in topological-insulator nanowires. *Nat. Commun.* **12**, 1–6 (2021).

[5] Legg, H. F., Loss, D. & Klinovaja, J. Majorana bound states in topological insulators without a vortex. *Phys. Rev. B* **104**, 165405 (2021).

[6] Köhler, H. Anisotropic g-factor of the conduction electrons in Bi_2Te_3. *Phys. Status Solidi (B)* **75**, 127–136 (1976).

[7] Ideue, T. *et al.* Bulk rectification effect in a polar semiconductor. *Nat. Phys.* **13**, 578–583 (2017).

[8] Wang, Y. *et al.* Gigantic magnetochiral anisotropy in the topological semimetal $ZrTe_5$. *arXiv:2011.03329* (2020).

[9] Borgwardt, N. *et al.* Self-organized charge puddles in a three-dimensional topological material. *Phys. Rev. B* **93**, 245149 (2016).

[10] Bömerich, T., Lux, J., Feng, Q. T. & Rosch, A. Length scale of puddle formation in compensation-doped semiconductors and topological insulators. *Phys. Rev. B* **96**, 075204 (2017).

[11] Knispel, T. *et al.* Charge puddles in the bulk and on the surface of the topological insulator $BiSbTeSe_2$ studied by scanning tunneling microscopy and optical spectroscopy. *Phys. Rev. B* **96**, 195135 (2017).

[12] Huang, Y. & Shklovskii, B. I. Disorder effects in topological insulator nanowires. *Phys. Rev. B* **104**, 054205 (2021).

[13] Yang, F. *et al.* Dual-gated topological insulator thin-film device for efficient fermi-level tuning. *ACS Nano* **9**, 4050–4055 (2015).

[14] Taskin, A. A. *et al.* Planar Hall effect from the surface of topological insulators. *Nat. Commun.* **8**, 1340 (2017).

[15] Bardarson, J. H., Brouwer, P. W. & Moore, J. E. Aharonov-bohm oscillations in disordered topological insulator nanowires. *Phys. Rev. Lett.* **105**, 156803 (2010).

[16] Zhang, Y. & Vishwanath, A. Anomalous Aharonov-Bohm conductance oscillations from topological insulator surface states. *Phys. Rev. Lett.* **105**, 206601 (2010).

[17] Cho, S. *et al.* Aharonov–Bohm oscillations in a quasi-ballistic three-dimensional topological insulator nanowire. *Nat. Commun.* **6**, 7634 (2015).

[18] Jauregui, L. A., Pettes, M. T., Rokhinson, L. P., Shi, L. & Chen, Y. P. Magnetic field-induced helical mode and topological transitions in a topological insulator nanoribbon. *Nat. Nano.* **11**, 345–351 (2016).

[19] Taskin, A. A., Sasaki, S., Segawa, K. & Ando, Y. Manifestation of topological protection in transport properties of epitaxial Bi_2Se_3 thin films. *Phys. Rev. Lett.* **109**, 066803 (2012).

[20] Rikken, G. L. J. A. & Wyder, P. Magnetoelectric anisotropy in diffusive transport. *Phys. Rev. Lett.* **94**, 016601 (2005).

[21] Pop, F., Auban-Senzier, P., Canadell, E., Rikken, G. L. J. A. & Avarvari, N. Electrical magnetochiral anisotropy in a bulk chiral molecular conductor. *Nat. Commun.* **5**, 3757 (2014).

[22] Krstić, V., Roth, S., Burghard, M., Kern, K. & Rikken, G. Magneto-chiral anisotropy in charge transport through single-walled carbon nanotubes. *J. Chem. Phys.* **117**, 11315–11319 (2002).

[23] He, P. *et al.* Observation of out-of-plane spin texture in a $SrTiO_3(111)$ two-dimensional electron gas. *Phys. Rev. Lett.* **120**, 266802 (2018).

[24] Nonlinear magnetotransport shaped by fermi surface topology and convexity. *Nat. Commun.* **10**, 1290 (2019).

[25] Guillet, T. *et al.* Observation of large unidirectional Rashba magnetoresistance in Ge(111). *Phys. Rev. Lett.* **124**, 027201 (2020).

[26] He, P. *et al.* Bilinear magnetoelectric resistance as a probe of three-dimensional spin texture in topological surface states. *Nat. Phys.* **14**, 495–499 (2018).

[27] Rikken, G. L. J. A. & Avarvari, N. Strong electrical magnetochiral anisotropy in tellurium. *Phys. Rev. B* **99**, 245153 (2019).

[28] Wakatsuki, R. *et al.* Nonreciprocal charge transport in noncentrosymmetric superconductors. *Sci. Adv.* **3**, e1602390 (2017).

[29] Qin, F. *et al.* Superconductivity in a chiral nanotube. *Nat. Commun.* **8**, 14465 (2017).

[30] Yasuda, K. *et al.* Nonreciprocal charge transport at topological insulator/superconductor interface. *Nat. Commun.* **10**, 2734 (2019).

[31] Baumgartner, C. *et al.* Supercurrent rectification and magnetochiral effects in symmetric Josephson junctions. *Nat. Nanotech.* (2021).

[32] Yasuda, K. *et al.* Large non-reciprocal charge transport mediated by quantum anomalous hall edge states. *Nat. Nanotech.* **15**, 831–835 (2020).

[33] Lustikova, J. *et al.* Vortex rectenna powered by environmental fluctuations. *Nat. Commun.* **9**, 4922 (2018).

[34] Avci, C. O., Mendil, J., Beach, G. S. D. & Gambardella, P. Origins of the unidirectional spin Hall magnetoresistance in metallic bilayers. *Phys. Rev. Lett.* **121**, 087207 (2018).

[35] Avci, C. O. *et al.* Unidirectional spin Hall magnetoresistance in ferromagnet/normal metal bilayers. *Nat. Phys.* **11**, 570–575 (2015).

[36] Li, T. *et al.* Origin of threshold current density for asymmetric magnetoresistance in Pt/Py bilayers. *Appl. Phys. Express* **10**, 073001–073001 (2017).

[37] Yin, Y. *et al.* Thickness dependence of unidirectional spin-Hall magnetoresistance in metallic bilayers. *Appl. Phys. Lett.* **111**, 232405 (2017).

[38] Lidig, C. *et al.* Unidirectional spin Hall magnetoresistance as a tool for probing the interfacial spin polarization of Co_2MnSi. *Phys. Rev. Applied* **11**, 044039 (2019).

[39] Olejník, K., Novák, V., Wunderlich, J. & Jungwirth, T. Electrical detection of magnetization reversal without auxiliary magnets. *Phys. Rev. B* **91**, 180402 (2015).

[40] Yokouchi, T. *et al.* Electrical magnetochiral effect induced by chiral spin fluctuations. *Nat. Commun.* **8**, 866 (2017).

[41] Lv, Y. *et al.* Unidirectional spin-Hall and Rashba-Edelstein magnetoresistance in topological insulator-ferromagnet layer heterostructures. *Nat. Commun.* **9**, 111 (2018).

[42] Yasuda, K. *et al.* Large unidirectional magnetoresistance in a magnetic topological insulator. *Phys. Rev. Lett.* **117**, 127202 (2016).

[43] Fan, Y. *et al.* Unidirectional magneto-resistance in modulation-doped magnetic topological insulators. *Nano Lett.* **19**, 692–698 (2019).

[44] Aoki, R., Kousaka, Y. & Togawa, Y. Anomalous nonreciprocal electrical transport on chiral magnetic order. *Phys. Rev. Lett.* **122**, 057206 (2019).
